# The Ku complex promotes DNA end-bridging and this function is antagonized by Tel1/ATM kinase

**DOI:** 10.1093/nar/gkad062

**Published:** 2023-02-10

**Authors:** Carlo Rinaldi, Paolo Pizzul, Erika Casari, Marco Mangiagalli, Renata Tisi, Maria Pia Longhese

**Affiliations:** Dipartimento di Biotecnologie e Bioscienze, Università degli Studi di Milano-Bicocca, 20126 Milano, Italy; Dipartimento di Biotecnologie e Bioscienze, Università degli Studi di Milano-Bicocca, 20126 Milano, Italy; Dipartimento di Biotecnologie e Bioscienze, Università degli Studi di Milano-Bicocca, 20126 Milano, Italy; Dipartimento di Biotecnologie e Bioscienze, Università degli Studi di Milano-Bicocca, 20126 Milano, Italy; Dipartimento di Biotecnologie e Bioscienze, Università degli Studi di Milano-Bicocca, 20126 Milano, Italy; Dipartimento di Biotecnologie e Bioscienze, Università degli Studi di Milano-Bicocca, 20126 Milano, Italy

## Abstract

DNA double-strand breaks (DSBs) can be repaired by either homologous recombination (HR) or non-homologous end-joining (NHEJ). NHEJ is induced by the binding to DSBs of the Ku70–Ku80 heterodimer, which acts as a hub for the recruitment of downstream NHEJ components. An important issue in DSB repair is the maintenance of the DSB ends in close proximity, a function that in yeast involves the MRX complex and Sae2. Here, we provide evidence that Ku contributes to keep the DNA ends tethered to each other. The *ku70-C85Y* mutation, which increases Ku affinity for DNA and its persistence very close to the DSB ends, enhances DSB end-tethering and suppresses the end-tethering defect of *sae2*Δ cells. Impairing histone removal around DSBs either by eliminating Tel1 kinase activity or nucleosome remodelers enhances Ku persistence at DSBs and DSB bridging, suggesting that Tel1 antagonizes the Ku function in supporting end-tethering by promoting nucleosome removal and possibly Ku sliding inwards. As Ku provides a block to DSB resection, this Tel1 function can be important to regulate the mode by which DSBs are repaired.

## INTRODUCTION

DNA double-strand breaks (DSBs) are particularly dangerous forms of damage that must be repaired to maintain genomic integrity. The two main mechanisms devoted to repair DNA DSBs are homologous recombination (HR), which uses intact homologous DNA as a template to restore the genetic information lost at the break site, and non-homologous end-joining (NHEJ), which catalyzes the direct religation of the DSB ends (1,2). Generation of DSBs also triggers activation of a checkpoint response, whose apical proteins include Mec1 (ATR in mammals) and Tel1 (ATM in mammals) kinases that couple DSB repair with cell-cycle progression (3).

Ku and MRX/MRN (Mre11–Rad50–Xrs2 in yeast; MRE11–RAD50–NBS1 in mammals) are among the first protein complexes to be recruited at DSBs (4). In both yeast and mammals, MRX/MRN activates the protein kinase Tel1/ATM that plays an important role in DSB signalling (5–7). Furthermore, MRX/MRN plays a critical role in initiating resection of the DSB ends to generate single-stranded DNA (ssDNA) that is the substrate for the recombinase Rad51 to catalyze homologous pairing and strand invasion (1). The current model for DSB resection posits that the Mre11 endonuclease catalyzes an endonucleolytic cleavage of the 5’-terminated strand at both the DSB ends, in a reaction stimulated by Sae2/CtIP phosphorylated by cyclin-dependent kinases (Cdk1 in yeast) (8–10). This step is followed by bidirectional resection from the nick, with Mre11 exonuclease resecting towards the DNA end and either Exo1 or Dna2 in conjunction with the helicase Sgs1 resecting away from the DSB (11–19). The absence of Sae2 leads to an increased MRX and Tel1 persistence at DSBs (20,21). Increased Tel1 signalling activity results in the accumulation of Rad9 close to the DSB ends and hyperactivation of the Rad53 kinase, which in turn inhibit Dna2-Sgs1- and Exo1-catalyzed end resection, respectively (22–26). DSB resection is also counteracted by the Ku complex, which is known to inhibit the resection activity of Exo1 by preventing its access to the DSB ends (27–33).

The Ku complex, which comprises the two Ku70 and Ku80 subunits, recognizes with avid affinity and no sequence specificity a large variety of DNA ends, including blunt ends, hairpin DNA, and ends with protruding single-stranded overhangs (34–36). Ku orthologs are found in organisms ranging from bacteria to humans (37,38). In eukaryotes, the two Ku subunits share three structural domains, consisting of an N-terminal von Willebrand A (vWA)-like domain, a central β-barrel domain, and an α-helical C-terminal arm. Structural analyses have revealed that Ku70 and Ku80 form a heterodimer that adopts a quasi-symmetric structure with a ring that encircles the duplex DNA (39,40). Furthermore, Ku binds DNA ends asymmetrically, with the Ku70 vWA-like domain facing outwards in close proximity to the DNA end and the Ku80 vWA-like domain facing inwards (40–42).

In addition of protecting the DSB ends from degradation by preventing the access of Exo1 to the DSB ends, the Ku complex acts as a hub to directly or indirectly recruit downstream NHEJ components (43). Canonical proteins involved in NHEJ in mammals include the DNA-dependent protein kinase catalytic subunit (DNA-PKcs) that interacts with Ku to form the DNA-PK holoenzyme, XRCC4 (Lif1 in yeast), XLF (Nej1 in yeast) and PAXX. The final NHEJ step relies on DNA ligase IV (Dnl4 in yeast), which forms a constitutive complex with XRCC4/Lif1 to ligate the DNA ends (44).

A central aspect of NHEJ is the maintenance of the DNA ends in close proximity to direct repair, a function that in *Saccharomyces cerevisiae* involves the MRX complex and Sae2 (45–49). Disparate conclusions have been drawn about the involvement of NHEJ proteins in this phenomenon. In fact, pull-down assays using either purified proteins or cell-free extracts have reported that Ku alone (50), DNA-PKcs alone (51), or the DNA-PK holoenzyme (52–56) could bridge the DNA ends. Furthermore, Ku and the XRCC4-DNA ligase IV complex have been found to be necessary and sufficient to mediate a flexible synapsis of two DNA ends, whereas either alone is not (57–59). Single-molecule FRET studies in *Xenopus* egg extracts showed that the NHEJ factors can assemble into a long-range complex, in which the DNA ends are laterally aligned and are held together by Ku, DNA-PKcs, DNA Ligase IV, XRCC4 and XLF (60–62). Upon dissociation of DNA-PKs, this high-order structural assembly is converted into an end-to-end close contact configuration, in which the DNA ends are aligned for ligation (60–62). On the other hand, a single molecule study of bacterial NHEJ, a mechanism relying on a homodimeric Ku and Ligase D, revealed that the Ku dimer alone is sufficient to form DNA bridges that are stabilized upon addition of Ligase D (63). This situation is similar to the NHEJ mechanism found in yeast cells, which do not possess DNA-PKcs, leaving open the possibility that Ku may be a central component in the formation of a molecular DSB bridge. Despite these observations, whether the Ku complex is important to support DSB end-tethering remains to be elucidated.

Here, we report the identification and characterization of the *S. cerevisiae ku70-C85Y* allele that, like *KU70* deletion, restores DNA damage resistance of *sae2*Δ cells. However, unlike *KU70* deletion that suppresses the resection defect of *sae2*Δ cells by relieving inhibition of Exo1 resection activity (30–32), the *ku70-C85Y* mutation suppresses the DSB end-tethering defect of *sae2*Δ cells, whereas the lack of Ku70 exacerbates it. The C85Y mutation, which increases Ku affinity for DNA, enhances Ku persistence closeness to the DSB end and decreases it at more distant sites. Suppression of *sae2*Δ end-tethering defect and increased Ku persistence very close to the DSB ends can also be observed when histone removal around a DSB is defective either by eliminating Tel1 kinase activity or nucleosome remodelers. Altogether, these findings lead to a model whereby Ku contributes to keeping the DNA ends tethered to each other, whereas Tel1 kinase antagonizes this Ku function by promoting histone removal around DSBs.

## MATERIALS AND METHODS

### Yeast strains and growth conditions

*S. cerevisiae* is the experimental model used in this study. Strain genotypes are listed in Supplementary Table S1. Strains JKM139, used to detect DSB resection, YMV45, used to detect DSB repair by SSA, and tGI354, used to detect ectopic recombination, were kindly provided by J. Haber (Brandeis University, Waltham, USA). Strains YJK40.6, used to detect end-tethering, was kindly provided by D. P. Toczyski (University of California, San Francisco, USA). Gene disruptions and tag fusions were generated by one-step PCR and standard yeast transformation procedures. Primers used for disruptions and gene tagging are listed in Supplementary Table S2. Cells were grown in YEP medium (1% yeast extract, 2% bactopeptone) supplemented with 2% glucose (YEPD), 2% raffinose (YEPR) or 2% raffinose and 3% galactose (YEPRG). All experiments were performed at 25°C.

### Search for *ku70* mutations that suppress the DNA damage sensitivity of *sae2*Δ cells

To search for *ku70* alleles that suppress the sensitivity of *sae2*Δ to phleomycin, genomic DNA from strains carrying the *URA3* gene located 500 bp upstream of the *KU70* ORF was used as a template to amplify by low-fidelity PCR the *KU70* coding region. Thirty independent PCR reaction mixtures were prepared, each containing 5U EuroTaq DNA polymerase (Euroclone), 10 ng genomic DNA, 500 ng each primer, 0.5 mM each dNTP (dATP, dTTP, dCTP), 0.1 mM dGTP, 0.5 mM MnCl_2_, 10 mM Tris–HCl (pH 8.3), 50 mM KCl, and 1.5 mM MgCl_2_. The resulting PCR amplification products, containing the *KU70* coding sequence and the *URA3* marker gene, were used to transform a *sae2*Δ strain. 3000 transformants were selected on synthetic medium without uracil and then assayed by drop tests for increased resistance to phleomycin compared to *sae2*Δ cells.

### DSB resection

YEPR exponentially growing cell cultures of JKM139 derivative strains, carrying the HO cut site at the *MAT* locus, were transferred to YEPRG at time zero. *Ssp*I-digested genomic DNA was run on an alkaline agarose gel and visualized after hybridization with an RNA probe that anneals with the unresected strand at one side of the HO-induced DSB (64). This probe was obtained by *in vitro* transcription using Promega Riboprobe System-T7 and plasmid pML514 as a template. Plasmid pML514 was constructed by inserting in the pGEM7Zf vector a 900-bp fragment containing part of the *MAT* locus (coordinates 200870 to 201587 on chromosome III). Quantitative analysis of DSB resection was performed by calculating the ratio of band intensities for ssDNA and the total amount of DSB products. The resection efficiency was normalized with respect to the HO cleavage efficiency for each time point. Densitometric analysis of band intensities was performed using Scion Image Beta 4.0.2 software.

### DSB repair by single-strand annealing (SSA) and ectopic recombination

DSB repair by SSA was detected in YMV45 derivative strains by Southern blot analysis using an *Asp*718-*Sal*I fragment containing part of the *LEU2* gene as a probe. Quantitative analysis of DSB repair was determined by calculating the ratio of band intensities for SSA to the total amount of SSA and DSB products for each time point. To normalize to cut efficiency, the value of the uncut band was subtracted from the total amount of SSA and DSB products. DSB repair by ectopic recombination was detected by using the tGI354 strain. To determine the repair efficiency, the intensity of the uncut band at 2 h after HO induction (maximum efficiency of DSB formation), normalized respect to a loading control, was subtracted to the normalized values of noncrossover (NCO) and crossover (CO) bands at the subsequent time points after galactose addition. The obtained values were divided by the normalized intensity of the uncut *MAT*a band at time zero before HO induction (100%). Densitometric analysis of band intensities was performed using Scion Image Beta 4.0.2 software.

### Plasmid religation assay

The centromeric pRS316 plasmid was digested with the *Bam*HI restriction enzyme before being transformed into the cells. Parallel transformation with undigested pRS316 DNA was used to determine the transformation efficiency. Efficiency of religation was determined by counting the number of colonies that were able to grow on medium selective for the plasmid marker and was normalized respect to the transformation efficiency for each sample. The religation efficiency in mutant cells was compared to that of wild-type cells that was set up to 100%.

### Southern blot analysis of telomere length

To determine the length of native telomeres, *Xho*I-digested genomic DNA was subjected to 0.8% agarose gel electrophoresis and hybridized with a ^32^P-labeled poly(GT) probe. Standard hybridization conditions were used.

### Western blotting

Protein extracts for western blot analysis were prepared by trichloroacetic acid (TCA) precipitation. Frozen cell pellets were resuspended in 100 μl 20% TCA. After the addition of acid-washed glass beads, the samples were vortexed for 10 min. The beads were washed with 200 μl of 5% TCA twice, and the extract was collected in a new tube. The crude extract was precipitated by centrifugation at 850 × *g* for 10 min. TCA was discarded and samples were resuspended in 70 μl 6 × Laemmli buffer (60 mM Tris pH 6.8, 2% SDS, 10% glycerol, 100 mM DTT, 0.2% bromophenol blue) and 30 μl 1 M Tris pH 8.0. Prior to loading, samples were boiled and centrifuged at 850 × *g* for 10 min. The supernatant containing the solubilized proteins was separated on 10% polyacrylamide gels. HA- and Myc-tagged proteins were detected by using anti-HA (12CA5) (1:2000) or anti-Myc (9E10) (1:1000) antibodies, respectively.

### Chromatin immunoprecipitation (ChIP) and quantitative PCR (qPCR)

YEPR exponentially growing cell cultures of JKM139 derivative strains, carrying the HO cut site at the *MAT* locus, were transferred to YEPRG at time zero. Crosslinking was done with 1% formaldehyde for 10 min (Exo1) or 15 min (Ku70, Ku70^C85Y^, H2A, and H3). The reaction was stopped by adding 0.125 M glycine for 5 min. Immunoprecipitation was performed by incubating samples with Dynabeads Protein G (ThermoFisher Scientific) for 3 h at 4 °C in the presence of 5 μg anti-HA (12CA5) or anti-Myc (9E10) antibodies. H2A and H3 histones were immunoprecipitated by using 5 μg anti‐H2A (39945, Active Motif) or 4 μg anti-H3 (ab1791, Abcam) antibodies. Quantification of immunoprecipitated DNA was achieved by qPCR on a Bio-Rad CFX Connect™ Real-Time System apparatus and Bio-Rad CFX Maestro 1.1 software. Triplicate samples in 20 μl reaction mixture containing 10 ng of template DNA, 300 nM for each primer, 2 × SsoFast™ EvaGreen supermix (Bio-Rad #1725201) (2 × reaction buffer with dNTPs, Sso7d-fusion polymerase, MgCl_2_, EvaGreen dye, and stabilizers) were run in white 96-well PCR plates Multiplate™ (Bio-Rad #MLL9651). The qPCR program was as follows: step 1, 98 °C for 2 min; step 2, 90 °C for 5 s; step 3, 60 °C for 15 s; step 4, return to step 2 and repeat 40 times. At the end of the cycling program, a melting program (from 65 to 95°C with a 0.5°C increment every 5 s) was run to test the specificity of each qPCR. Data are expressed as fold enrichment at the HO-induced DSB over that at the non-cleaved *ARO1* locus, after normalization of the ChIP signals to the corresponding input for each time point. Fold enrichment was then normalized to the efficiency of DSB induction. For histone loss, the fold enrichment from each sample after HO induction was divided by the fold enrichment from uninduced cells, and log_2_ of the resulting values was calculated. Oligonucleotides used for qPCR analyses are listed in Supplementary Table S3.

### Coimmunoprecipitation

Total protein extracts were prepared by breaking cells in 400 μl of buffer containing 50 mM HEPES pH 7.5, 300 mM NaCl, 20% glycerol, 1 mM sodium orthovanadate, 60 mM β‐glycerophosphate, and protease inhibitor cocktail (Roche Diagnostics). An equal volume of breaking buffer was added to clarified protein extracts and tubes were incubated for 2 h at 4 °C with 50 μl of Protein G-Dynabeads and 5 μg anti-Flag (F1804 by Sigma) antibodies. The resins were then washed twice with 1 mL of breaking buffer. Bound proteins were visualized by western blotting with an anti-Flag (F1804 by Sigma) (1:500) or an anti-HA (12CA5) (1:2000) antibody after electrophoresis on a 10% SDS-polyacrylamide gel.

### Recombinant production and purification of Ku heterodimers

The bicistronic constructs containing Ku70-Strep or Ku70^C85Y^-Strep and Ku80-6xHis were designed as previously described (65). In particular, the genes were chemically synthetized (GenScript, Piscataway, NJ, USA) and cloned into the pET21a vector. Ku heterodimers were recombinantly produced in *Escherichia coli* BL21 (DE3) cells and purified. Briefly, the Ku70–Ku80 and Ku70^C85Y^–Ku80 heterodimers were produced in ZYM-5052 medium (66) supplemented with ampicillin (100 mg/L), extracted and purified by immobilized ion metal affinity chromatography (ABT, Torrejon de Ardoz, Madrid, Spain), followed by Strep purification on Strep-Tactin resin (IBA Lifesciences, Gottingen, Germany). Fractions containing the highest amount of protein were pooled and buffer-exchanged with HEPES buffer (HEPES 25 mM, NaCl 100 mM, pH 7) by gel filtration on PD-10 columns (GE Healthcare, Little Chalfont, UK). Protein concentration was determined with the Bradford assay (Bio-Rad, Hercules, USA), using bovine serum albumin as a standard. SDS-PAGE was performed on 12% polyacrylamide gels and stained with Gel-Code Blue (Pierce, Rockford, USA) after electrophoresis. Broad-range, pre-stained molecular-mass markers (GeneSpin, Milan, Italy) were used as standards.

### Size-exclusion chromatography (SEC) analysis

The quaternary structure of Ku70–Ku80 and Ku70^C85Y^–Ku80 was determined by SEC with an NGC Quest 10 Plus Chromatography System (Bio-Rad, California, USA), equipped with a Superdex 200 10/30 column (GE Healthcare, Little Chalfont, UK) with a cutoff of 10–600 kDa. Chromatographic separations were carried out in HEPES buffer (HEPES 25 mM, NaCl 100 mM, pH 7) as the mobile phase, at a flow rate of 0.5 ml/min and a protein concentration of 0.5 mg/ml; chromatograms were recorded at 280 nm. The molecular weight was determined using a calibration curve made with the following standards: M-βGal (450.93 kDa), yeast alcohol dehydrogenase (150 kDa), BSA (66.5 kDa), Lipase B of *Candida antarctica* (34.7 kDa), green fluorescence protein (27.5 kDa), and cytochrome *c* (horse heart, 12.4 kDa). For each standard protein the distribution coefficient (*K*_*d*_) was calculated using the following equation:

$$\begin{equation*}{\rm{\;}}{K_d} = \;\frac{{{V_E} - {V_0}}}{{{V_T} - {V_0}}}\end{equation*}$$

where *V_E_* is the elution volume, *V_0_* is the void volume, which is determined with blue dextran (2000 kDa), and *V_T_* is the total volume, determined with Uracil (0.112 kDa). The calibration curve Log(MW) versus *K_d_* was built and the interpolated linear equation was used to calculate Ku70–Ku80 and Ku70^C85Y^–Ku80 molecular weight from their *K_d_* values. Experiments were performed in triplicate.

### Electrophoretic mobility shift assay (EMSA)

EMSA was performed by incubating 13 nM of 21 bp ^32^P-labeled dsDNA (5’‐CCGCACACCCACACACCAGTG‐3’) with purified Ku70–Ku80 and Ku70^C85Y^–Ku80 (0; 26; 39; 52; 78; 104 nM) in ice for 30 min in binding buffer (100 mM NaCl, 25 mM Tris/HCl pH 7.5, 2 mM MgCl_2_, 7% (v/v) glycerol and 1 mM DTT) to a final volume of 50 μl. Reactions were loaded on a non-denaturing 6% acrylamide/bisacrylamide gel and separated by running for 4 hours at 120 V at 4 °C using a low‐ionic strength buffer (6.73 mM Tris–HCl pH 7.5, 3.3 mM NaOAc pH 5, 1 mM EDTA). The gel was soaked for 15 min in 10% methanol, 10% acetic acid solution and vacuum-dried.

### Molecular modeling

The structural models for Ku70^C85Y^ mutant protein within the Ku70–Ku80 heterodimer were prepared starting from the crystal structure (PDB ID: 5Y58). PDB file was processed with MAESTRO (Schrödinger Release 2022-2: Maestro, Schrödinger, LLC, New York, NY, 2021) using the Protein Preparation Wizard tool (67) to add missing hydrogen atoms and assign proper bond orders, and with PRIME (68) to fill in missing loops and side chain atoms. The mutation was generated in MAESTRO replacing the original side chains with the mutated residue tool. The regions in a range of 10 Å from the mutation were minimized using MACROMODEL (Schrödinger Release 2022-2: MacroModel, Schrödinger, LLC, New York, NY, 2021). Minimizations were carried on using AMBER force field (69) with implicit solvent, using PRCG method with maximum iteration of 2500 and a gradient convergence threshold of 0.05. In order to investigate the interaction of Ku70–Ku80 and Ku70^C85Y^–Ku80 heterodimer with DNA, the structure of a Ku-bound DNA (sequence 5’-TAAACTAAAAAC-3’) was extracted from the crystal of the human Ku complex (PDB ID: 1JEY). The DNA end was blunted by removal of the protruding end and submitted as a binding partner to HADDOCK2.4 server (70) together with Ku70–Ku80 or Ku70^C85Y^–Ku80 heterodimer. The interface was defined by a constraint for active residues comprising the conserved positive residues facing the β-barrel in each heterodimer, such as R73, R265, R298 and K333 for Ku70 and R41, R210, R258, K401 and K402 for Ku80. The best position for DNA within the Ku–DNA complex was obtained with the Ku70^C85Y^–Ku80 complex, and adopted for the wild-type complex as well. The HADDOCK2.4 refinement protocol (71) was used to refine the models of wild-type Ku70–Ku80 and Ku70^C85Y^–Ku80 heterodimers with the DNA molecule described above, with the minimized energy protocol with standard parameters (https://wenmr.science.uu.nl/haddock2.4/settings#refinement). Briefly, this minimization protocol performs a series of short molecular dynamics (MD) simulations with explicit solvent after a solvent shell is built around the complex with position restraint on the α backbone of the protein, thus allowing the amino acids chains to move. Next, 1250 MD steps are performed at 300 K with position restraints for residues not involved in intermolecular contacts within 5 Å. Finally, the system is cooled down (1000 MD steps at 300, 200 and 100 K) with position restraints on the heavy atoms of the protein complex, excluding the interface atoms. The protocol output is the energetically lowest conformation for the complex and the corresponding HADDOCK score, which is an indicator of the interaction strength (HADDOCK-score_itw_ = 1.0*E_vdw_ + 0.2*E_elec_ + 1.0*E_desolv_ + 0.1*E_air_, where *E*_vdw_ is the van der Waals energy, *E*_elec_ is the electrostatic energy, *E*_desolv_ is the desolvation energy and *E*_air_ is the restraint violation energy, which is not added in refinement since no ambiguous interaction restraints are imposed).

### Statistical analysis

Statistical analysis was performed using Microsoft Excel Professional 365 software. *P-*values were determined by using an unpaired two-tailed *t*-test. No statistical methods or criteria were used to estimate the size or to include or exclude samples.

## RESULTS

### Identification of *ku70* alleles that suppress the DNA damage sensitivity of *sae2*Δ cells

**Figure 1.**
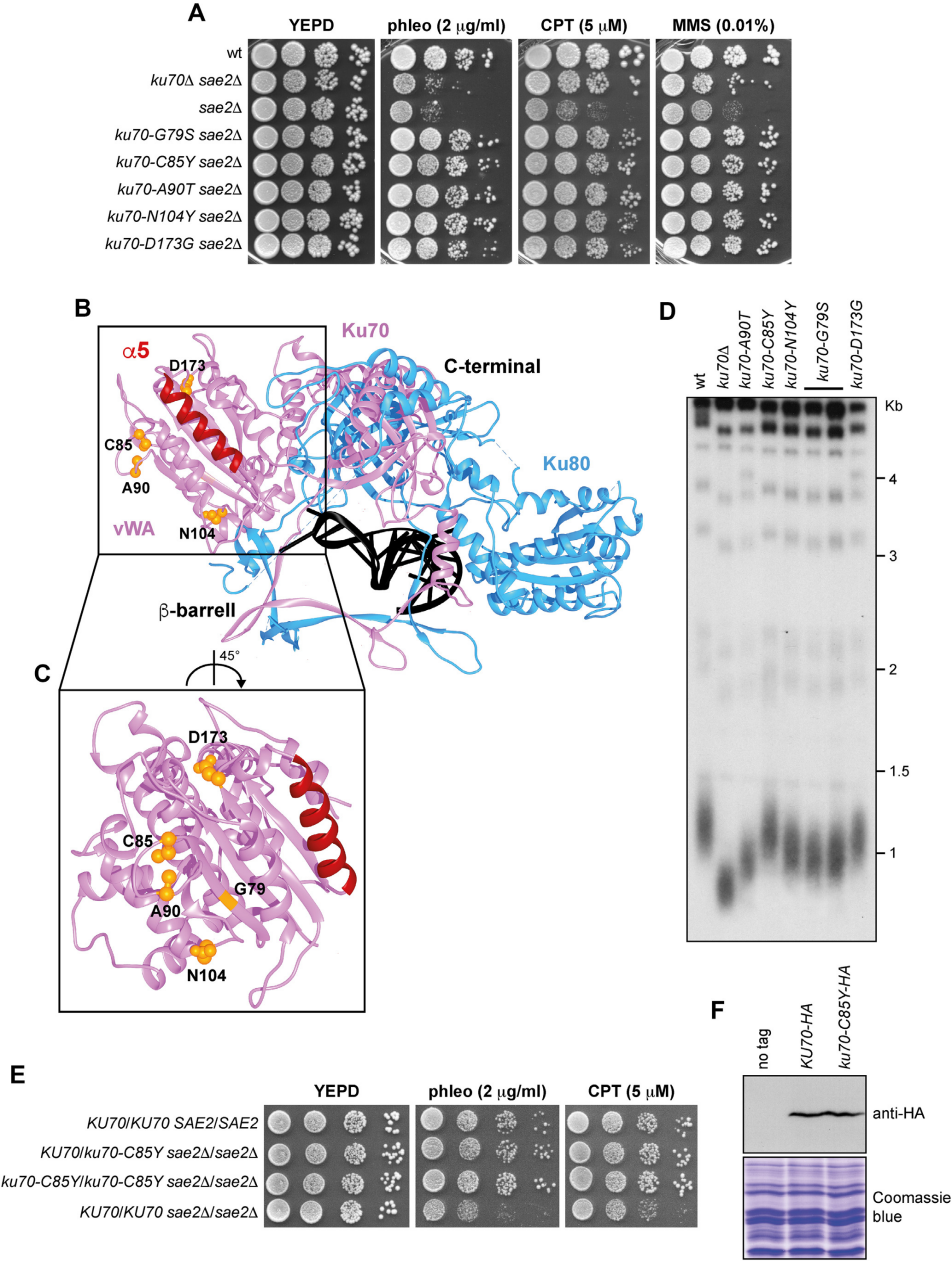
Identification of *ku70* alleles that suppress the sensitivity to phleomycin of *sae2*Δ cells. (**A**) Exponentially growing cultures were serially diluted (1:10) and each dilution was spotted out onto YEPD plates with or without phleomycin (phleo), camptothecin (CPT) or methyl methanesulfonate (MMS). (**B**) The structure of yeast Ku70–Ku80 heterodimer (PDB ID: 5Y58) is shown as a cartoon. Ku70 is in pink (with α5 helix in red), Ku80 in blue, and the nucleic acid in black. The residues affected by the mutations are shown as orange balls. (**C**) The structure of Ku70 vWA-like domain is shown in detail with α5 helix in red. The residues affected by the mutations are shown as orange balls. (**D**) Telomere length. *Xho*I-cut genomic DNA from exponentially growing cells was subjected to Southern blot analysis using a radiolabeled poly(GT) telomere-specific probe. (**E**) Exponentially growing cultures were serially diluted (1:10) and each dilution was spotted out onto YEPD plates with or without phleomycin or CPT. (**F**) Western blot with an anti-HA antibody of protein extracts. The same amount of protein extracts was separated on a SDS-PAGE and stained with Coomassie blue as loading control.

The lack of Sae2, which leads to hypersensitivity to DNA damaging agents, impairs resection and tethering of DNA DSB ends (48). Deletion of *KU70* suppressed the sensitivity to camptothecin (CPT) and methyl methanesulfonate (MMS) of *sae2*Δ cells by relieving inhibition of Exo1 nuclease (30–32), whereas it conferred no rescue of sensitivity to phleomycin (Figure 1A). We have previously identified *mre11* mutations that increase resistance of *sae2*Δ cells to phleomycin and suppress their end-tethering but not their resection defect (72), suggesting that end-tethering can be particularly important to repair phleomycin-induced DNA lesions. To understand the contribution of DSB end-tethering in DNA damage resistance and the possible role of Ku in this phenomenon, we searched for *ku70* mutations that restored the resistance to phleomycin of *sae2*Δ cells. *KU70* gene was amplified by low-fidelity PCR, followed by transformation into *sae2*Δ cells with linear *KU70* PCR products in order to replace the corresponding *KU70* wild-type sequence with the mutagenized DNA fragments. Transformants were then screened for increased viability in the presence of phleomycin compared to *sae2*Δ cells. This analysis allowed us to identify the *ku70-G79S*, *ku70-C85Y*, *ku70-A90T*, *ku70-N104Y*, and *ku70-D173G* alleles. These mutations restored DNA damage resistance of *sae2*Δ cells not only to phleomycin but also to CPT and MMS, whereas, as expected, *ku70*Δ was effective only in the suppression of *sae2*Δ CPT and MMS sensitivities (Figure 1A).

The Ku70 and Ku80 subunits share a similar topology. Their structure is composed of three domains: an N-terminal α/β domain similar to a vWA domain, a β-barrel with the function of enclosing the nucleic acid, and a helical C-terminal arm that leans towards the vWA-like domain of the other subunit (40) (Figure 1B). The identified Ku70 mutations are all localized in the conserved N-terminal vWA-like domain. None of the substitutions generates critical problems to the protein structure according to an analysis with Missense 3D suite (73) and, with the exception of A90 and N104, many of the residues are buried inside the structure. In detail, both C85Y and A90T mutations introduce a bulkier amino acid (with a reduction in the surrounding cavity volume of 31.752 and 9.288 Å^3^, respectively) and target the same β-hairpin structure, which is localized in the nearby region of the conserved α5 helix that was shown to be involved in Ku–Ku self-association in humans (74) (Figure 1B and C). The N104 resides in a loop exposed to solvent immediately adjacent to the same β-hairpin and the N104Y substitution leads to exposition of an aromatic amino acid. The G79S and D173G mutations affect residues localized in the embedded β-sheet within the vWA-like domain, with G79 being nearby the 84–93 amino acid β-hairpin and substituting a buried G residue with a bulkier amino acid, while D173 forming a saline bridge with K213 that is lost in the D173G mutant (Figure 1B and C). None of the residues is in conserved vWA traits and none of the substitutions is likely to disrupt the domain structure, although all of them are non-conservative substitutions.

The lack of Ku affects the length of telomeres by destabilizing the interaction between the telomerase subunit Est1 and the telomeric DNA (75–77). We found that the *ku70-G79S*, *ku70-A90T*, and *ku70-N104Y* alleles shortened telomeres although not severely as *ku70*Δ, whereas both *ku70-C85Y* and *ku70-D173G* did not (Figure 1D). As the C85Y mutation does not affect telomere length, we focused the analysis on this mutation.

The ability of *ku70-C85Y* to suppress the sensitivity of *sae2*Δ to genotoxic agents was dominant, as *KU70*/*ku70-C85Y* *sae2*Δ/*sae2*Δ diploid cells were less sensitive to phleomycin and CPT compared to *KU70*/*KU70* *sae2*Δ/*sae2*Δ diploid cells (Figure 1E), suggesting that *ku70-C85Y* allele encodes an hypermorphic variant. The mutation did not alter protein level, as similar amount of Ku70 was detected in protein extracts from wild-type and *ku70-C85Y* cells (Figure 1F).

### The *ku70-C85Y* allele does not suppress the resection defect and checkpoint hyperactivation of *sae2*Δ cells

The lack of Sae2 enhances MRX/Tel1 signalling activity that leads to an increased Rad9 association with DSBs and hyperactivation of the downstream checkpoint kinase Rad53 that causes a persistent cell-cycle arrest (20,21). The increased Rad9 binding at DSBs acts as a barrier to Sgs1-Dna2-mediated DSB resection (22–24,26), whereas Rad53 hyperactivation results in inhibitory phosphorylation of Exo1 (25), thus accounting for the *sae2*Δ resection defect. Both the resection defect and persistent checkpoint activation contribute to the DNA damage hypersensitivity of *sae2*Δ cells. In fact, deletion of *KU70* partially suppresses both the DNA damage sensitivity and the resection defect of *sae2*Δ cells because inhibition of Exo1 resection activity is relieved (30–32). Furthermore, dampening checkpoint activation either by reducing MRX/Tel1 binding to DSBs or by impairing Rad53 or Tel1 kinase activity restores DNA damage resistance and DSB resection of *sae2*Δ cells (24,26,72,78,79).

**Figure 2.**
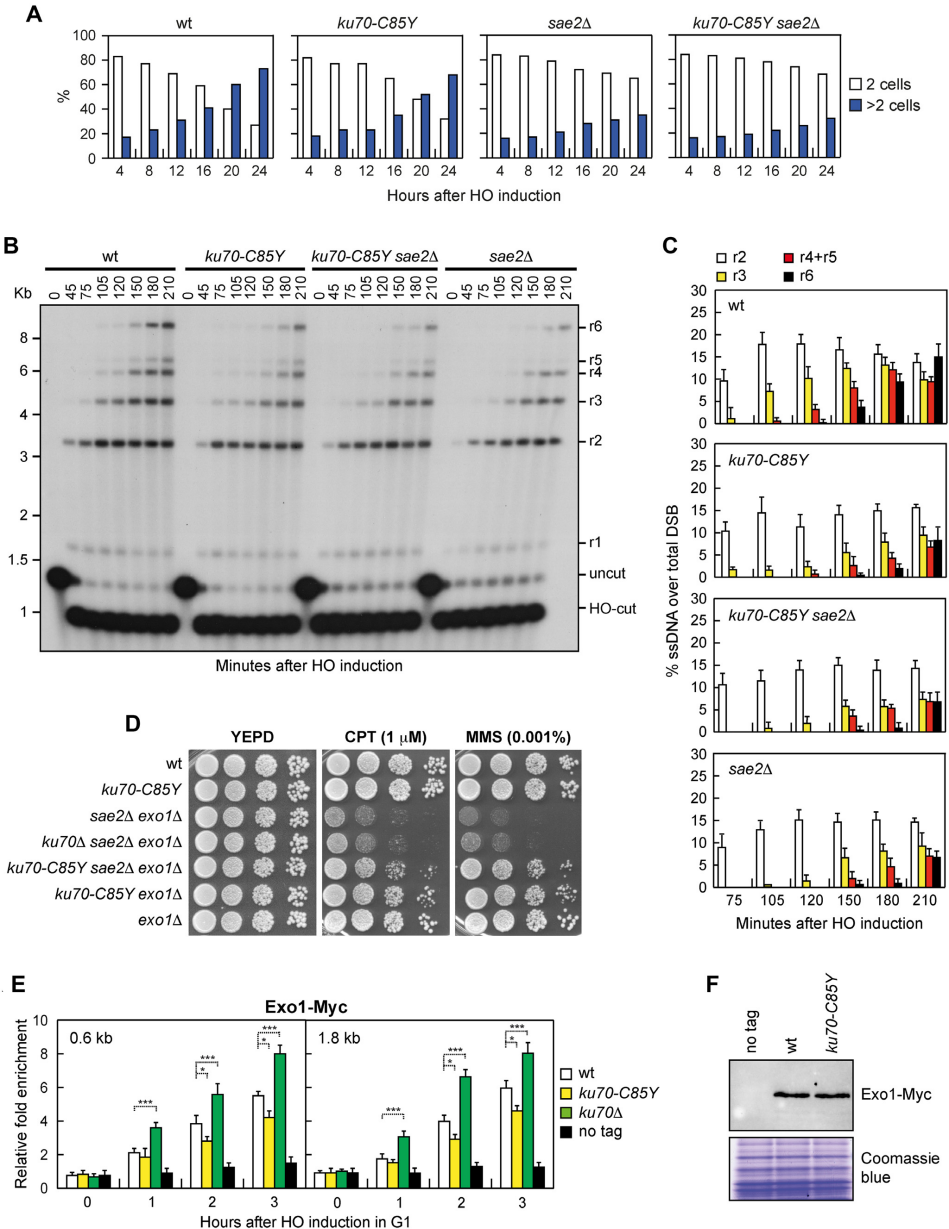
Suppression of *sae2*Δ DNA damage sensitivity by *ku70-C85Y* does not involve Exo1 and DSB resection. (**A**) YEPR G1-arrested cell cultures of JKM139 derivative strains, carrying the HO cut site at the *MAT* locus, were plated on galactose-containing plates (time zero). 200 cells for each strain were analyzed to determine the frequency of cells forming microcolonies with two cells (2 cells) and of more than two cells (>2 cells). (**B**) DSB resection. JKM139 derivative strains were transferred to YEPRG at time zero. *Ssp*I-digested genomic DNA was hybridized with a single-stranded *MAT* probe that anneals with the unresected strand. 5’-3’ resection produces *Ssp*I fragments (r1 through r6) detected by the probe. (**C**) Densitometric analysis of the resection products. The mean values of three independent experiments as in (B) are represented with error bars denoting standard deviation (s.d.). (**D**) Exponentially growing cultures were serially diluted (1:10) and each dilution was spotted out onto YEPD plates with or without CPT or MMS. (**E**) ChIP and qPCR. YEPR G1-arrested cell cultures of JKM139 derivative strains were transferred to YEPRG to induce HO in the presence of α-factor. Relative fold enrichment of Exo1-Myc at the indicated distances from the HO cleavage site was determined after ChIP with an anti-Myc antibody and qPCR. The mean values of three independent experiments are represented with error bars denoting s.d. ***p< 0.005; *p< 0.05 (unpaired two-tailed Student's *t*-test). (**F**) Western blot with an anti-Myc antibody of extracts used for the ChIP analysis shown in (E). The same amounts of extracts were separated on a SDS-PAGE and stained with Coomassie blue as loading control.

To assess the mechanism underlying suppression by the *ku70-C85Y* allele, first we investigated whether the *ku70-C85Y* allele can partially suppress the DNA damage sensitivity of *sae2*Δ cells by dampening checkpoint activation. To measure checkpoint activation, we used a haploid JKM139 strain background that expresses the site-specific *HO* endonuclease gene from a galactose-inducible promoter (80). In this strain, induction of HO upon galactose addition leads to the generation of a single DSB at the *MAT* locus that cannot be repaired by HR because the homologous donor loci *HML* and *HMR* are deleted. When G1-arrested cell cultures were spotted on galactose-containing plates to induce HO, most wild-type, *ku70-C85Y*, *sae2*Δ, and *ku70-C85Y sae2*Δ cells arrested as large budded cells within 4 hours after HO induction (Figure 2A), indicating that the checkpoint is activated. A checkpoint response triggered by a single unrepairable DSB can be turned off, allowing cells to resume cell-cycle progression through a process called adaptation (81,82). The enhanced checkpoint activation in *sae2*Δ cells prevents cells from adapting to the checkpoint triggered by an unrepaired DSB (20,21). We found that wild-type and *ku70-C85Y* cells were capable to adapt to the checkpoint and to form microcolonies with more than 2 cells within 24 h, whereas most *sae2*Δ and *ku70-C85Y sae2*Δ cells remained arrested at the 2-cell dumbbell stage (Figure 2A). Thus, we can conclude that *ku70-C85Y* does not suppress the DNA damage sensitivity of *sae2*Δ cells by decreasing checkpoint activation triggered by an unrepaired DSB.

Next, we asked whether the *ku70-C85Y* allele behaves like *ku70*Δ and therefore restores DNA damage resistance of *sae2*Δ cells by suppressing their resection defect. To monitor DSB resection we used JKM139 derivative strains, where a single irreparable DSB at the *MAT* locus can be induced by HO expression. Because ssDNA cannot be cleaved by restriction enzymes, ssDNA generation was assessed by testing resistance to cleavage as resection proceeds beyond the *Ssp*I restriction site located at different distances from the HO-cut site. *Ssp*I-resistant ssDNA can be detected as appearance of slower migrating bands (r1-r6) after denaturing gel electrophoresis of *Ssp*I-digested genomic DNA and hybridization with a probe that anneals to the unresected strand at one side of the DSB (Supplementary Figure S1). As expected, *sae2*Δ cells showed a resection defect of the HO-induced DSB that was similar to that of *ku70-C85Y sae2*Δ cells (Figure 2B and C), indicating that *ku70-C85Y* does not suppress the DNA damage sensitivity of *sae2*Δ cells by restoring DSB resection.

Removal of Ku70 suppresses the DNA damage sensitivity and the resection defect of *sae2*Δ cells in an Exo1-dependent manner (30–32). Consistent with different suppression mechanisms by the *ku70*Δ and *ku70-C85Y* alleles, *ku70*Δ failed to suppress the sensitivity to CPT and MMS of *sae2*Δ *exo1*Δ cells, whereas *ku70-C85Y sae2*Δ *exo1*Δ cells were more resistant to CPT and MMS than *sae2*Δ *exo1*Δ cells (Figure 2D), indicating that Exo1 is not required for *sae2*Δ suppression by *ku70-C85Y*. Similar results have been obtained with the other *G79S*, *A90T*, *N104Y* and *D173G* mutations (Supplementary Figure S2A). Furthermore, while deletion of *KU70* suppressed the sensitivity to CPT and MMS of cells expressing the nuclease defective *mre11-H125N* allele and restored viability of *sae2*Δ *sgs1*Δ cells, *ku70-C85Y* did not (Supplementary Figure S2B and C), suggesting that the C85Y mutation bypasses a Sae2 function that does not involve Sgs1 and DSB resection.

Interestingly, while *KU70* deletion did not affect DSB resection (28), *ku70-C85Y* cells showed a resection defect compared to wild-type cells (Figure 2B and C). As Ku limits DSB resection by inhibiting the recruitment of Exo1 to DSBs (31), we analyzed the effect of the *ku70-C85Y* mutation on Exo1 association with DSBs. To minimize the effect of DSB resection on protein binding to DSBs, HO was induced in G1-arrested cells that were kept arrested in G1 with α-factor throughout the experiment. In fact, the low Cdk1 activity in G1 cells prevents resection of the HO-induced DSB (83,84). Consistent with previous observations (31), chromatin immunoprecipitation (ChIP) and quantitative PCR (qPCR) showed that the lack of Ku70 increased Exo1 association with the HO-induced DSB (Figure 2E). By contrast, although protein extract from wild-type and *ku70-C85Y* cells contained a similar amount of Exo1 (Figure 2F), *ku70-C85Y* cells showed a decreased Exo1 association with the HO-induced DSB compared to wild-type cells (Figure 2E). This finding indicates that the *ku70-C85Y* allele encodes a hypermorphic Ku70 variant that limits Exo1 association with the DSB ends more efficiently than wild-type Ku70.

### The *ku70-C85Y* allele suppresses the end-tethering defect of *sae2*Δ cells

**Figure 3.**
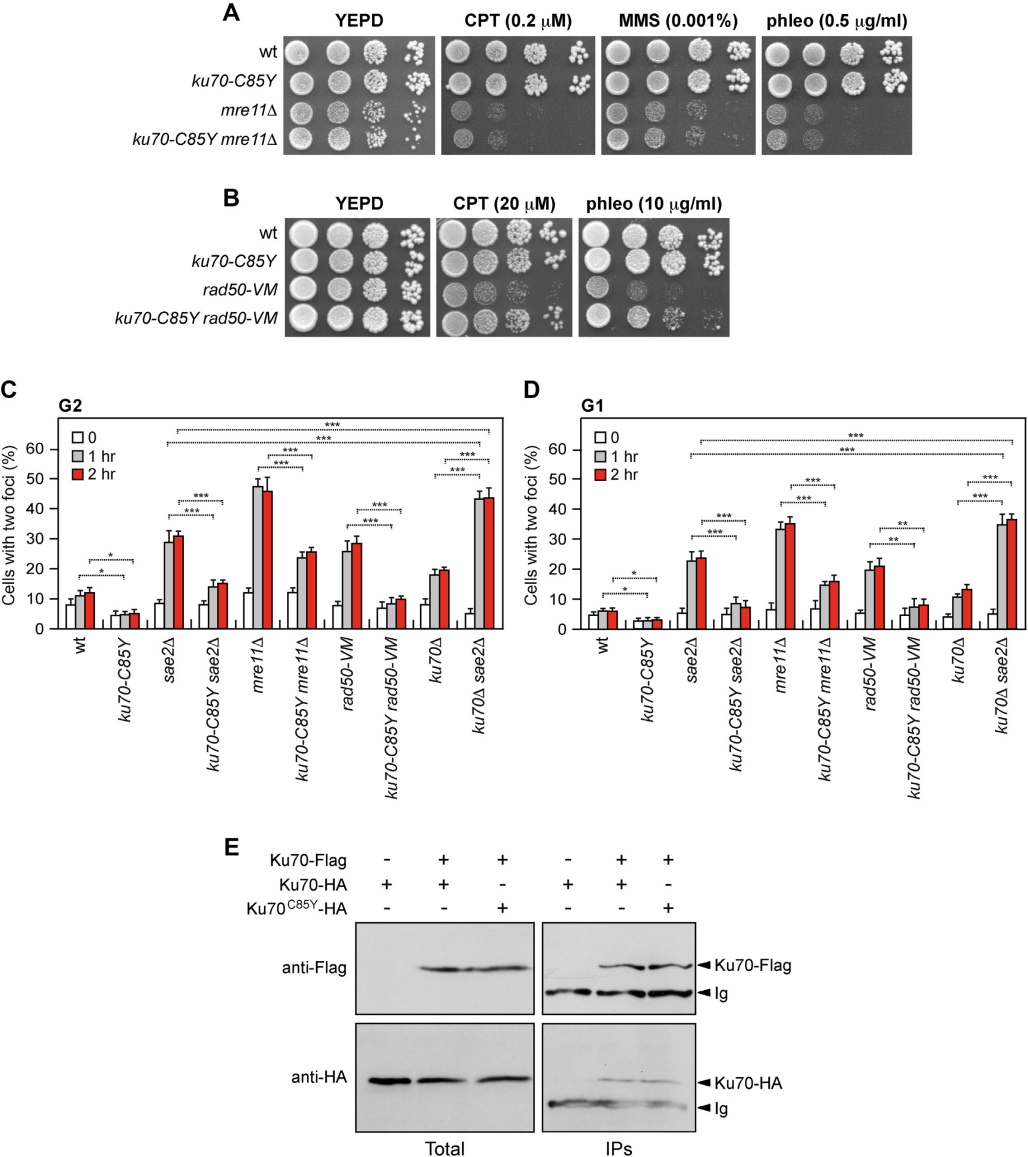
The *ku70-C85Y* allele suppresses the end-tethering defect of *sae2*Δ cells. (**A, B**) Exponentially growing cultures were serially diluted (1:10) and each dilution was spotted out onto YEPD plates with or without CPT, MMS or phleomycin. (**C**, **D**) DSB end-tethering. Exponentially growing YEPR cell cultures were arrested in G2 with nocodazole (**C**) or in G1 with α-factor (**D**) at time zero and transferred to YEPRG in the presence of nocodazole or α-factor, respectively. 200 cells for each strain were analyzed to determine the percentage of cells showing two LacI-GFP foci. The mean values of three independent experiments are represented with error bars denoting s.d. ***p< 0.005; **p <0.01; *p< 0.05 (unpaired two-tailed Student's *t*-test). (**E**) Ku70 can self associate. Protein extracts were analyzed by western blotting with an anti-Flag or an anti-HA antibodyeither directly (Total) or after immunoprecipitation (IPs) with an anti-Flag antibody.

We investigated the effect of *ku70-C85Y* on the DNA damage sensitivity of cells lacking Mre11 or expressing the *rad50-VM* allele, which encodes a Rad50 mutant variant that specifically impairs the tethering of the DSB ends (85). The *ku70-C85Y* allele failed to suppress the severe DNA damage sensitivity of *mre11*Δ cells (Figure 3A), whereas it was capable of partially restoring DNA damage resistance of *rad50-VM* cells (Figure 3B). This result, together with the observation that Sae2 is involved in keeping the DSB ends tethered to each other (47,48), raises the possibility that the *ku70-C85Y* allele can suppress the DNA damage sensitivity of *sae2*Δ cells by restoring DSB end-tethering.

To visualize DNA regions flanking to an HO-induced DSB, we used a strain background where multiple repeats of the LacI repressor binding site are integrated 50 kb upstream and downstream of an irreparable HO break site located on chromosome VII in cells constitutively expressing a LacI-GFP fusion protein (45). The level of end-tethering upon DSB formation was determined by measuring the generation of one or two LacI-GFP foci. HO expression was induced by galactose addition to cell cultures that were arrested in G2 with nocodazole and kept blocked in G2 by nocodazole in order to ensure that all cells would arrest in metaphase. Consistent with an end-tethering defect (48), *sae2*Δ cells showed an increase of two LacI-GFP foci compared to the uninduced condition, whereas ∼90% of wild-type cells showed a single LacI-GFP focus 1–2 h after HO induction (Figure 3C). Strikingly, *sae2*Δ cells harboring the *ku70-C85Y* allele showed a decrease in the percentage of two LacI-GFP foci (Figure 3C), indicating that this mutation suppresses the end-tethering defect caused by the lack of Sae2. A slight but significant decrease of two LacI-GFP foci can be detected also in *ku70-C85Y* cells compared to wild-type cells (Figure 3C), suggesting that the Ku70^C85Y^ mutant variant possesses by itself an increased ability to support DSB end-tethering. Consistent with a role of the Ku complex in DSB end-tethering, the lack of Ku70 slightly increased the percentage of untethered ends after HO induction and this percentage was further increased in *ku70*Δ *sae2*Δ cells compared to each single mutant (Figure 3C).

The effect of *ku70*Δ and *ku70-C85Y* alleles on the frequency of LacI-GFP foci after HO induction was primarily due to end-tethering and not to cohesion defects. In fact, similar results have been obtained when HO was induced in α-factor-arrested cells that were kept arrested in G1 in the presence of galactose (Figure 3D).

The function of Ku in DSB end-tethering appears to be independent of MRX, as *ku70-C85Y* decreased the amount of two LacI-GFP foci of both *mre11*Δ and *rad50-VM* cells (Figure 3C and D). While the severe DNA damage hypersensitivity of *mre11*Δ cells, which was not suppressed by the *ku70-C85Y* allele (Figure 3A), is due to the lack of MRX functions in several aspects of the DNA damage response, *rad50-VM* cells are specifically defective in DSB tethering (85). Therefore, the increased DNA damage resistance of *ku70-C85Y rad50-VM* cells compared to *rad50-VM* cells (Figure 3B) can be due to the suppression of their DSB tethering defect.

A role for Ku in DSB end-bridging predicts that two Ku70–Ku80 heterodimers can interact. Consistent with this hypothesis, atomic force microscopy showed that DNA-bound human Ku can self-associate (52) and an interaction between two Ku heterodimers has been observed by coimmunoprecipitation in human cells (74). To assess a possible interaction between Ku70–Ku80 heterodimers in yeast, we performed coimmunoprecipitation using protein extracts prepared from diploid cells carrying differentially tagged versions of Ku70. We detected Ku70-HA in Ku70-Flag immunoprecipitates from both wild-type and *ku70-C85Y* protein extracts (Figure 3E). As neither Ku70 nor Ku80 homodimerizes (35,86,87), these results are consistent with differentially tagged Ku heterodimers forming multimers.

### The *ku70-C85Y* allele suppresses the HR defects of *sae2*Δ cells

**Figure 4.**
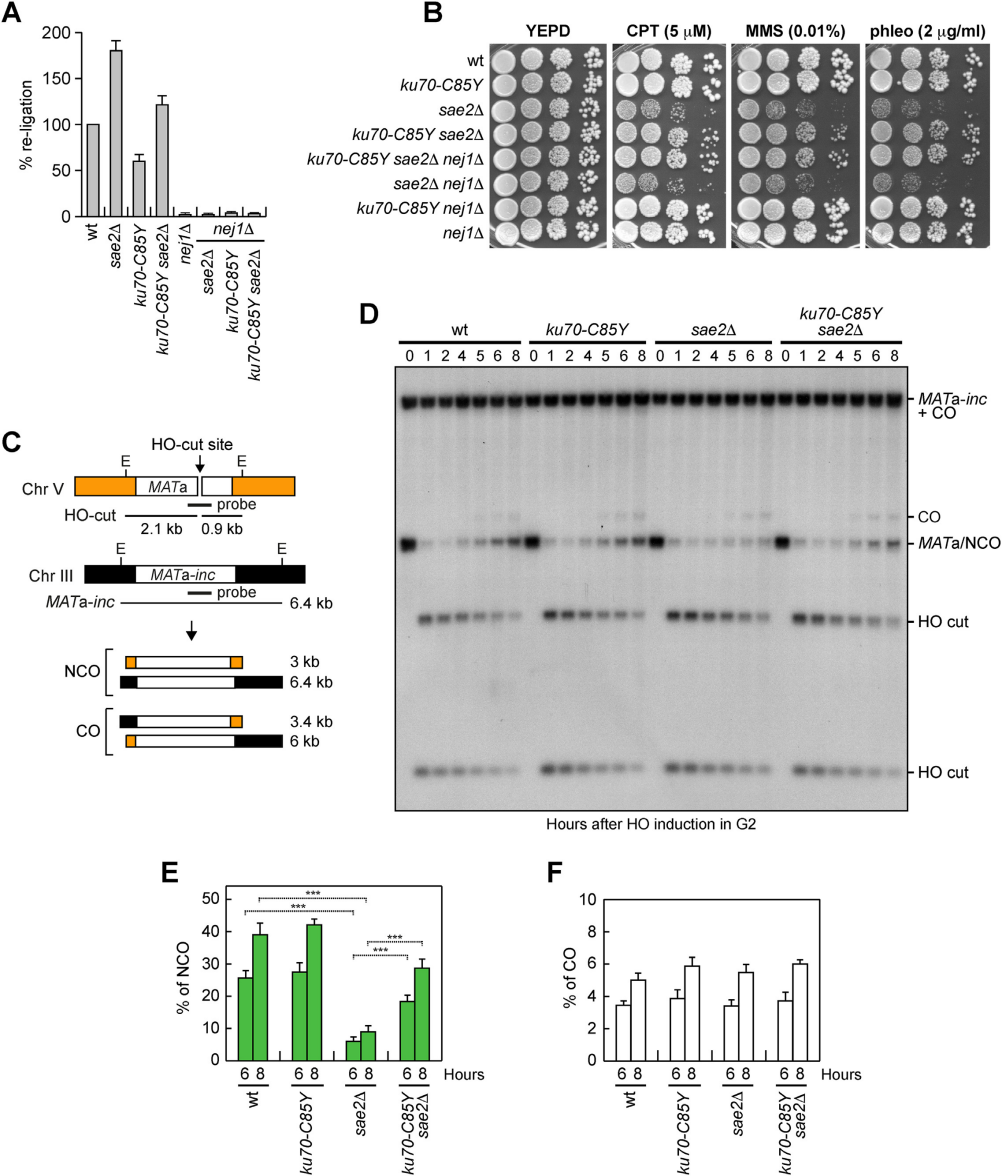
The *ku70-C85Y* allele suppresses the SDSA defect of *sae2*Δ cells. (**A**) Plasmid religation assay. The same amounts of *Bam*HI-linearized or uncut pRS316 plasmid DNA were transformed into the cells. Data are expressed as percentage of religation relative to wild type that was set up at 100% after normalization to the corresponding transformation efficiency of the uncut plasmid. (**B**) Exponentially growing cultures were serially diluted (1:10) and each dilution was spotted out onto YEPD plates with or without CPT, MMS or phleomycin. (**C–F**) DSB repair by ectopic recombination. (**C**) System to detect ectopic recombination. HO induction generates a DSB at a *MAT*a DNA sequence on chromosome V, while the homologous *MAT*a*-inc* region on chromosome III cannot be cut by HO and is used as a donor for HR-mediated repair that generate noncrossover (NCO) and crossover (CO) products. E, *Eco*RI. (**D**) YEPR cell cultures were arrested in G2 with nocodazole and transferred to YEPRG at time zero in the presence of nocodazole. Southern blot analysis of *Eco*RI-digested genomic DNA with a *MAT*a probe. (**E, F**) Densitometric analysis of NCO (**E**) and CO (**F**) band signals. The mean values of three independent experiments are represented with error bars denoting s.d. ***p< 0.005 (unpaired two-tailed Student's *t*-test).

The maintenance of the DSB ends tethered to each other can be important to repair a DSB by both NHEJ and HR. As DSB repair by NHEJ is increased in *sae2*Δ cells possibly due to the reduced DSB resection (72), it is unlikely that the restored end-tethering in *ku70-C85Y sae2*Δ cells leads to DNA damage resistance by increasing the efficiency of NHEJ. In fact, when we measured DSB repair by NHEJ as the ability of cells to religate a plasmid that was linearized before being transformed into the cells, *sae2*Δ cells, as expected, showed an increase in the efficiency of plasmid religation compared to wild-type cells (Figure 4A). By contrast, *ku70-C85Y* cells decreased it either in the presence or in the absence of Sae2 (Figure 4A), indicating that the *ku70-C85Y* mutation impairs DSB end-joining. Furthermore, the *ku70-C85Y* allele still suppressed the DNA damage sensitivity of *sae2*Δ cells lacking the NHEJ component Nej1 (Figure 4B), whose loss leads to end-joining defects (88,89), indicating that NHEJ is not required for *sae2*Δ suppression.

In the canonical HR pathway, the 3’-ended ssDNA tail at one of the DSB ends invades the homologous template, forming a D-loop structure that primes DNA synthesis. Then, the complementary sequence on the second end of the DSB can anneal to the displaced ssDNA to generate a double Holliday junctions, whose random cleavage yields to noncrossover (NCO) and crossover (CO) products. Alternatively, if the newly synthesized strand is unwound by the D-loop, its annealing with the 3’ ssDNA end at the other side of the DSB leads to the generation of NCO products in a process called synthesis-dependent strand-annealing (SDSA). Finally, when homology is present only for one end of the DSB, this single end invades the template and initiates extensive DNA synthesis that can reach even the end of the chromosome in a process called break‐induced replication (BIR) (Supplementary Figure S3) (1).

We have previously shown that the lack of Sae2 impairs DSB repair by SDSA (72). Furthermore, *mre11* mutations that suppressed the end-tethering defect of *sae2*Δ cells also suppressed their SDSA defect (72). Thus, we investigated whether the increased DNA damage resistance of *sae2*Δ cells conferred by the *ku70-C85Y* mutation might be due to a more efficient DSB repair by SDSA. To monitor CO and NCO formation, we used a haploid strain that carries a *MAT*a gene on chromosome V that can be cleaved by HO and repaired by using a *MAT*a (*MAT*a*-inc*) sequence on chromosome III that contains a single base pair substitution that prevents HO cleavage (90) (Figure 4C). Galactose was added to induce HO and then it was maintained in the medium to cleave the HO sites that were eventually reconstituted by NHEJ. As expected (72), the 3 kb *MAT*a band resulting from NCO recombination events reaccumulated less efficiently in *sae2*Δ cells compared to wild-type cells (Figure 4D and E), while the 3.4 kb band resulting from CO recombination events was similar in wild-type and *sae2*Δ cells (Figure 4D and F). The presence of the *ku70-C85Y* mutation, which did not affect by itself the generation of both COs and NCOs events, increased the NCO products in *sae2*Δ cells (Figure 4D–F). As most NCO products arise from the SDSA mechanism, this finding indicates that the *ku70-C85Y* allele suppresses the SDSA defect of *sae2*Δ cells.

**Figure 5.**
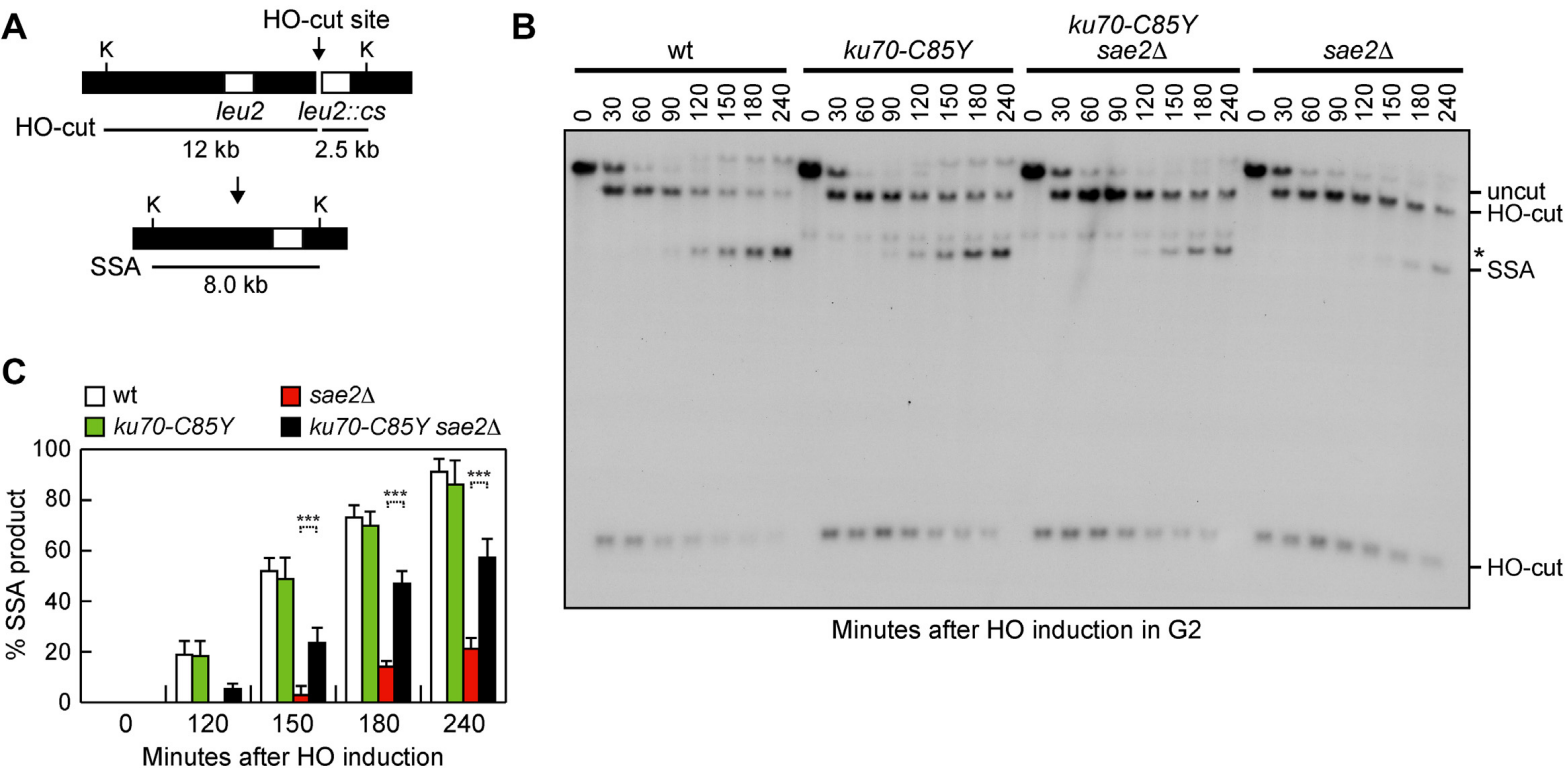
The *ku70-C85Y* allele suppresses the SSA defect of *sae2*Δ cells. (**A**) System to detect DSB repair by SSA. HO induction generates a DSB between two homologous *leu2* sequences that are 4.6 kb apart. K, *Kpn*I. (**B**) YEPR cell cultures were arrested in G2 with nocodazole and transferred to YEPRG at time zero in the presence of nocodazole. Southern blot analysis of *Kpn*I-digested genomic DNA with a *LEU2* probe revealed a 2.5 kb and 12 kb DNA fragments (HO-cut) resulting from HO-induced DSB formation. DSB repair by SSA generates an 8 kb fragment (SSA). *indicates cross-hybridization. (**C**) Densitometric analysis of the SSA band signals. The mean values of three independent experiments are represented with error bars denoting s.d. ***p< 0.005 (unpaired two-tailed Student's *t*-test).

A DSB that is flanked by direct repeats can be repaired by single-strand annealing (SSA), which requires resection of the direct repeats followed by annealing of the resulting complementary ssDNA (1). We have previously shown that the lack of Sae2 impairs DSB repair by SSA and that this SSA defect cannot be solely explained by the reduced DSB resection (48), raising the possibility that the end-tethering defect displayed by *sae2*Δ cells can contribute to the poor SSA efficiency. To investigate the effect of the *ku70-C85Y* allele on DSB repair by SSA, we used a strain carrying tandem repeats of the *LEU2* gene, with a recognition site for HO adjacent to one of the repeats (91) (Figure 5A). Galactose was added to G2-arrested cells to induce HO expression and it was maintained in the medium to recleave the HO sites reconstituted by NHEJ. We found that *sae2*Δ cells reduced the accumulation of the SSA repair products compared to wild-type cells, whereas the SSA repair events increased in *ku70-C85Y sae2*Δ cells (Figure 5B and C), indicating that *ku70-C85Y* suppresses the SSA defect caused by the lack of Sae2.

### The C85Y mutation increases Ku70 association with the DSB ends

**Figure 6.**
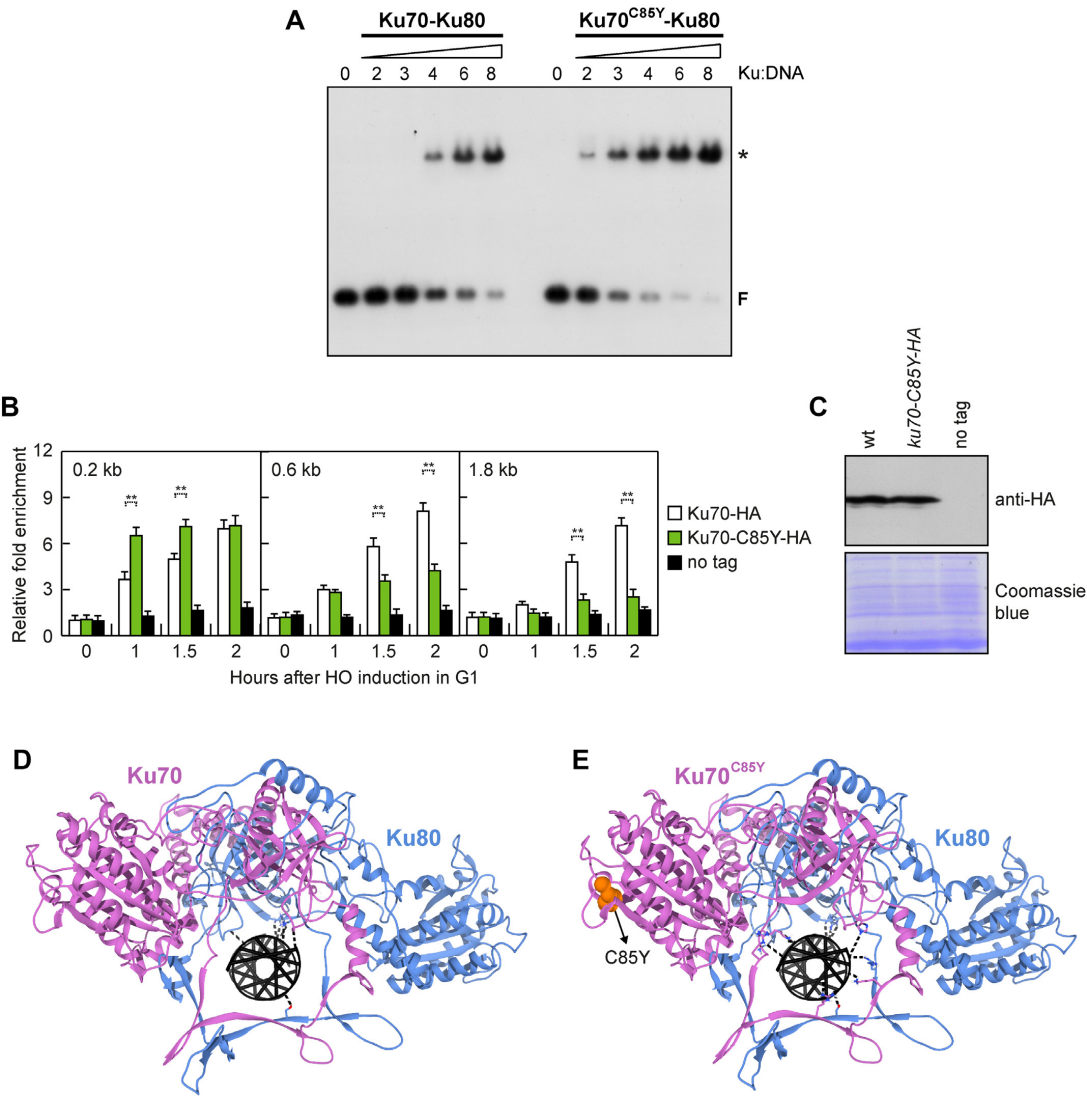
Ku association with DNA. (**A**) EMSA with a 21 bp dsDNA and increasing concentrations of Ku70–Ku80 and Ku70^C85Y^–Ku80 complexes. Bands corresponding to free DNA (F) and to a protein-DNA complex (asterisk) are denoted. (**B**) ChIP and qPCR. Exponentially growing YEPR cell cultures of JKM139 derivative strains were arrested in G1 with α-factor and transferred to YEPRG to induce HO in the presence of α-factor. Relative fold enrichment of Ku70-HA and Ku70^C85Y^-HA at the indicated distances from the HO cleavage site was determined after ChIP with an anti-HA antibody and qPCR. The mean values of three independent experiments are represented with error bars denoting s.d. **p <0.01 (unpaired two-tailed Student's *t*-test). (**C**) Western blot with an anti-HA antibody of extracts used for the ChIP analysis shown in (B). The same amount of protein extracts was separated on a SDS-PAGE and stained with Coomassie blue as loading control. (**D, E**) Structure of a Ku heterodimer bound to DNA was obtained by docking protocol on HADDOCK2.4 platform as described in the Materials and Methods section. The complexes of wild-type Ku70–Ku80 (**D**) or Ku70^C85Y^–Ku80 (**E**) bound to DNA, minimized by HADDOCK refinement protocol, are shown. Ku70 is in pink, Ku80 in blue, DNA in black. Residues making electrostatic contacts with DNA are exposed in sticks and the bounds are in dashed lines. The residue affected by the C85Y mutation in (E) is shown as orange balls.

The C85Y mutation might facilitate the tethering of the DSB ends either by increasing Ku–Ku self-interaction or by stabilizing Ku70 association with the DSB end. As we failed to detect an increase in Ku70–Ku70 interaction by coimmunoprecipitation in *ku70-C85Y* cells (Figure 3E), wild-type and mutant Ku protein complexes were tested for DNA-binding by gel electrophoretic mobility shift assay (EMSA). Both Ku70–Ku80 and Ku70^C85Y^–Ku80 heterodimers were produced and purified from *E. coli* cells to homogeneity (Supplementary Figure S4A) and tested for the ability to bind a 21 bp blunt-ended double-stranded DNA (dsDNA) that is capable of binding one Ku heterodimer (36). SEC analyses on purified complexes showed that Ku70–Ku80 and Ku70^C85Y^–Ku80 were dimers (Supplementary Figure S4B), indicating that the C85Y substitution does not affect the complex quaternary structure. Increasing concentrations of Ku heterodimer were added to a fixed amount of radiolabeled dsDNA substrate. The addition of either wild-type Ku70–Ku80 or Ku70^C85Y^–Ku80 complex resulted in one shifted band, likely representing DNA bound to one Ku dimer (Figure 6A). This single-bound species became detectable at a 4-fold ratio of wild-type Ku70–Ku80 to DNA substrate and at a 2-fold ratio of Ku70^C85Y^–Ku80 to DNA substrate (Figure 6A), indicating that the C85Y mutation increases the DNA binding property of the Ku heterodimer.

Next, we performed ChIP analysis to assess the consequences of the C85Y mutation on Ku70 association with DSBs. Consistent with an increased DNA binding propensity of Ku70^C85Y^–Ku80 (Figure 6A), the amount of Ku70^C85Y^ bound very closely to the HO-induced DSB (0.2 kb) was increased compared to wild-type Ku70 (Figure 6B), although similar amount of Ku70 and Ku70^C85Y^ can be detected in the protein extracts used for the ChIP analysis (Figure 6C). Interestingly, as the distance from the DSB increased (0.6 and 1.8 kb), the amount of Ku70^C85Y^ bound to the HO-induced DSB was lower than wild-type Ku70 (Figure 6B). As the Ku complex has been proposed to slide along DNA (92), this finding suggests that the increased DNA affinity of the mutant Ku70^C85Y^–Ku80 complex for DNA ends can result in a decreased propensity of the complex to diffuse over the broken DNA end and an increased ability to support the tethering of the DSB ends. Consistent with this hypothesis, the *ku70-Y494N* mutation, which was identified as a suppressor of the CPT sensitivity of *mre11* nuclease mutants, was shown to reduce DNA binding of the Ku heterodimer and to increase its probability to slide inwards once bound to dsDNA (93). Interestingly, the *ku70-Y494N* allele, which was capable of partially suppressing the CPT sensitivity of *sae2*Δ cells in an Exo1-dependent manner because of its failure to inhibit Exo1, did not suppress the phleomycin sensitivity of *sae2*Δ cells (Supplementary Figure S5A). Furthermore, it exacerbated the DNA damage sensitivity and the end-tethering defect of *rad50-VM* cells (Supplementary Figure S5B and C), thus supporting the hypothesis that the ability of Ku to support DSB end-tethering is influenced by its DNA binding properties.

In order to investigate the interaction of wild-type and mutant Ku heterodimers with a DNA end, and since no structure is available for yeast Ku bound to DNA, we performed an integrative docking software HADDOCK2.4 simulation for both wild-type Ku70–Ku80 and mutant Ku70^C85Y^–Ku80 heterodimers versus a 13 bp dsDNA molecule. Only with the Ku70^C85Y^–Ku80 heterodimer the docking protocol explored far different conformations and was able to position the DNA molecule inside the β-barrel, similarly to what observed in the human Ku complex with DNA (40). In order to obtain a wild-type Ku–DNA complex as well, we built a homology modeling structure of the wild-type heterodimer bound to DNA with the mutant heterodimer structure as a model. The models obtained were refined by the HADDOCK2.4 refinement protocol (71), which exploits molecular dynamics simulation in water solvent allowing freedom of movement first to the residues side chains all over the protein and then to the region of the protein taking contact with the interactor. The simulations generated structures with minimized energy and HADDOCK scores of -118.5 for the wild-type and -124.3 for the mutant heterodimer, mainly due to a higher number of electrostatic interactions in the mutant complex (the specific scores for electrostatic interactions being -548.7 versus -516.8 for Ku70^C85Y^–Ku80 versus Ku70–Ku80). By analyzing the structures, we could observe that the ring of the wild-type heterodimer is able to make only few electrostatic contacts with the DNA molecule (Figure 6D), as it was actually described in the available structures for the human Ku70–Ku80 complex (40). It is interesting to notice that the positive residues of the Ku70^C85Y^–Ku80 ring appeared to be able to make more salt-bridges and H-bonds with the DNA (Figure 6E), suggesting that the β-barrel of Ku70^C85Y^–Ku80 could be more flexible than that of the wild-type Ku70–Ku80, thus allowing the positive residues to orient their side chains towards the DNA molecule.

### Tel1 kinase antagonizes Ku function in DSB end-tethering

**Figure 7.**
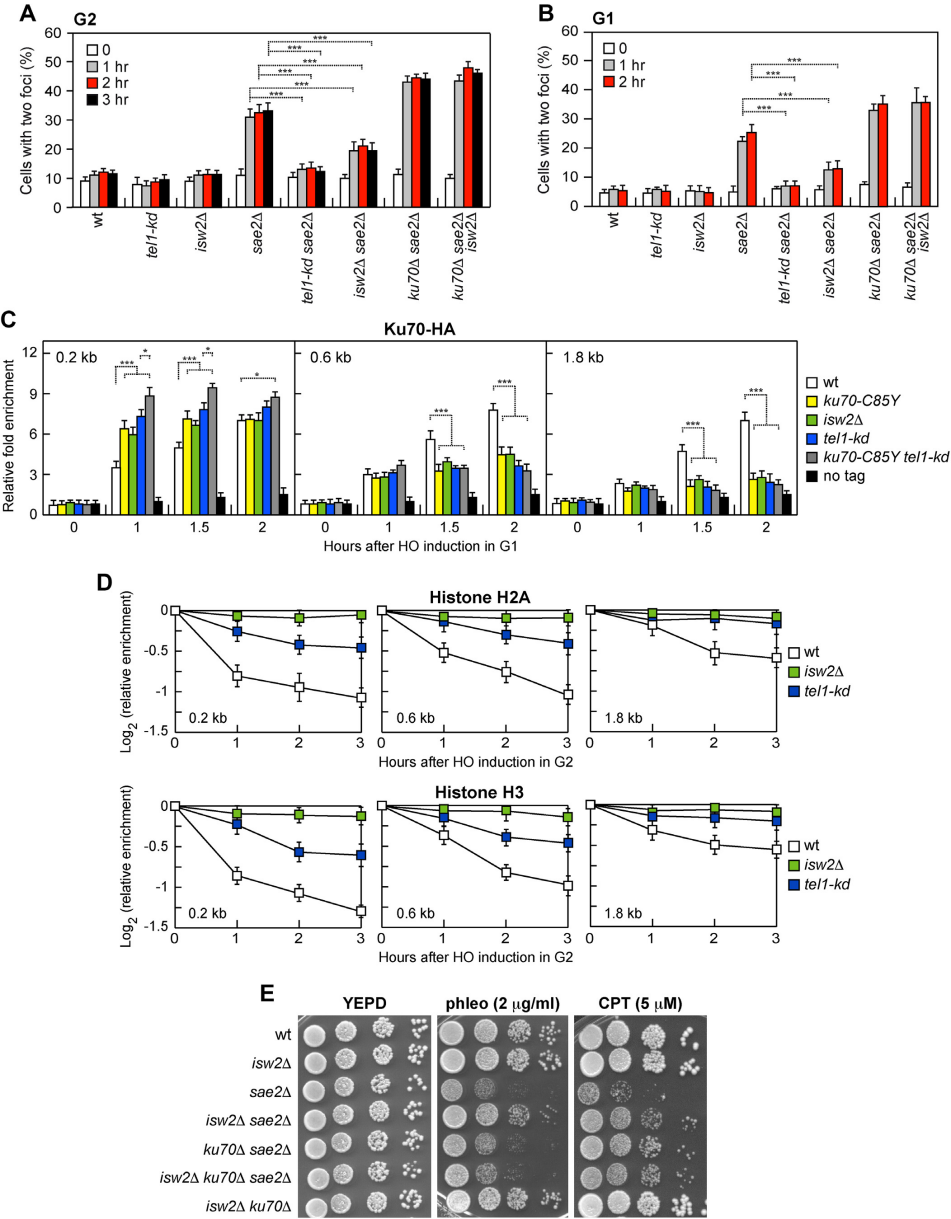
The lack of Tel1 kinase or of Isw2 suppresses the end-tethering defect of *sae2*Δ cells and increases Ku persistence close to the DSB ends. (**A, B**) DSB end-tethering. Exponentially growing YEPR cell cultures were arrested in G2 with nocodazole (**A**) or in G1 with α-factor (**B**) at time zero and transferred to YEPRG in the presence of nocodazole or α-factor, respectively. 200 cells for each strain were analyzed to determine the percentage of cells showing two LacI-GFP foci. The mean values of three independent experiments are represented with error bars denoting s.d. ***p<0.005 (unpaired two-tailed Student's *t*-test). (**C**) ChIP and qPCR. Exponentially growing YEPR cell cultures of JKM139 derivative strains were arrested in G1 with α-factor and transferred to YEPRG to induce HO in the presence of α-factor. Relative fold enrichment of Ku70-HA at the HO-induced DSB was evaluated after ChIP with an anti-HA antibody and qPCR. The mean values of three independent experiments are represented with error bars denoting s.d. ***p <0.005; *p< 0.05 (unpaired two-tailed Student's *t*-test). (**D**) ChIP and qPCR. HO expression was induced at time zero by galactose addition to G2-arrested cells that were kept arrested in G2 by nocodazole throughout the experiment. Relative fold enrichment of H2A or H3 at the HO-induced DSB was evaluated after ChIP with an anti-H2A or an anti-H3 antibody and qPCR analysis. The mean values of three independent experiments are represented with error bars denoting s.d. (**E**) Serial dilutions of exponentially growing cultures onto YEPD plates with or without CPT or phleomycin.

The lack of Tel1 increases Ku persistence very close to the DSB ends (94). Furthermore, mammalian ATM antagonizes tethering at single-ended DSBs (95), raising the possibility that Tel1/ATM can modulate DSB tethering by regulating Ku association with DNA. However, the possible role of Tel1 in regulating Ku-mediated end-tethering can be masked by the fact that Tel1 has a structural role in promoting MRX persistence at DSBs and therefore the lack of Tel1 impairs end-tethering by decreasing the amount of MRX bound at DSBs (85,96). As Tel1 increases MRX retention at DSBs independently of its kinase activity (85), we analyzed DSB end-tethering and Ku association with DSBs in cells expressing a Tel1 kinase defective (*tel1-kd*) allele (97), which has been already reported to suppress the DNA damage sensitivity of *sae2*Δ cells (24). We detected a significant decrease in percentage of two LacI-GFP foci in *tel1-kd sae2*Δ cells compared to *sae2*Δ cells (Figure 7A and B), indicating that Tel1 antagonizes DNA bridging. Furthermore, similar to Ku70^C85Y^, the lack of Tel1 kinase activity increased the amount of Ku70 bound in close proximity to the HO-induced DSB ends and this effect was more pronounced in the presence of the Ku70^C85Y^ mutant variant (Figure 7C). The amount of Ku70 bound at more distant sites (0.6 and 1.8 kb) from the HO-induced DSB was much lower in *tel1-kd* cells compared to wild type (Figure 7C), indicating that Tel1 controls Ku persistence at the DSB ends.

### Nucleosome removal from DSBs antagonizes Ku function in DSB end-tethering

Previous work has shown that Ku diffusion is inefficient on nucleosome-associated DNA ends (98). Furthermore, Ku and phosphorylated histone H2AX (γH2AX) foci are mutually exclusive (99), suggesting that Ku localizes to DNA ends that are locally depleted of nucleosomes. ChIP experiments support nucleosome disassembly near DSBs (100), with histone loss promoted by mammalian ATM (101,102). These findings lead to the hypothesis that Tel1 can control Ku spreading by promoting histone disassembly around a DSB. Thus, we evaluated the effect of the lack of Tel1 kinase activity on histone H2A and H3 occupancy centromere‐proximal to the irreparable HO-induced DSB at the *MAT* locus. HO expression was induced by galactose addition to G2‐arrested cells that were kept arrested in G2 with nocodazole to exclude possible effects of DNA replication on histone association with DNA. As expected, H2A and H3 signals near the HO-induced DSB decreased in wild-type cells, while they remained high in *tel1-kd* cells (Figure 7D), indicating that Tel1 kinase promotes nucleosome loss from DSBs.

If Tel1 antagonizes the Ku function in supporting DSB end-bridging by promoting histone removal from DSBs and Ku sliding inwards, failure to remove histones should mimic the effect caused by the lack of Tel1 kinase activity on DNA end-tethering and Ku association with DSBs. The density of nucleosome packaging is regulated by ATP-dependent chromatin remodelers, which use the energy derived from ATP hydrolysis to evict, assemble, reposition or exchange histones throughout the genome. We have previously shown that the lack of the chromatin remodeler Isw2 dramatically impairs nucleosome disassembly at DSBs (103), prompting us to test the effect of its deletion on *sae2*Δ suppression, Ku association with DSBs, and DSB tethering. The lack of Isw2, which impaired H2A and H3 removal from the HO-induced DSB (Figure 7D), partially suppressed both the DNA damage sensitivity (Figure 7E) and the end-tethering defect of *sae2*Δ cells (Figure 7A and B). Furthermore, similar to both *ku70-C85Y* and *tel1-kd*, *isw2*Δ increased Ku70 association very close to the HO-induced DSB end, whereas it decreased it at more distant sites (Figure 7C).

Suppression of both the DNA damage sensitivity and the end-tethering defect of *sae2*Δ cells by *ISW2* deletion requires Ku70. In fact, *isw2*Δ failed to suppress the phleomycin sensitivity of *ku70*Δ *sae2*Δ cells and did not further increase resistance to CPT of *ku70*Δ *sae2*Δ cells (Figure 7E). Furthermore, *isw2*Δ did not restore end-tethering of *ku70*Δ *sae2*Δ cells (Figure 7A and B). Unfortunately, the effect of the *tel1-kd* mutation on *ku70*Δ *sae2*Δ cells cannot be tested due to the senescence phenotype of *ku70*Δ *tel1-kd* cells (75). Altogether, these data indicate that histone removal from DSBs antagonizes the Ku function in supporting DSB bridging.

## DISCUSSION

**Figure 8.**
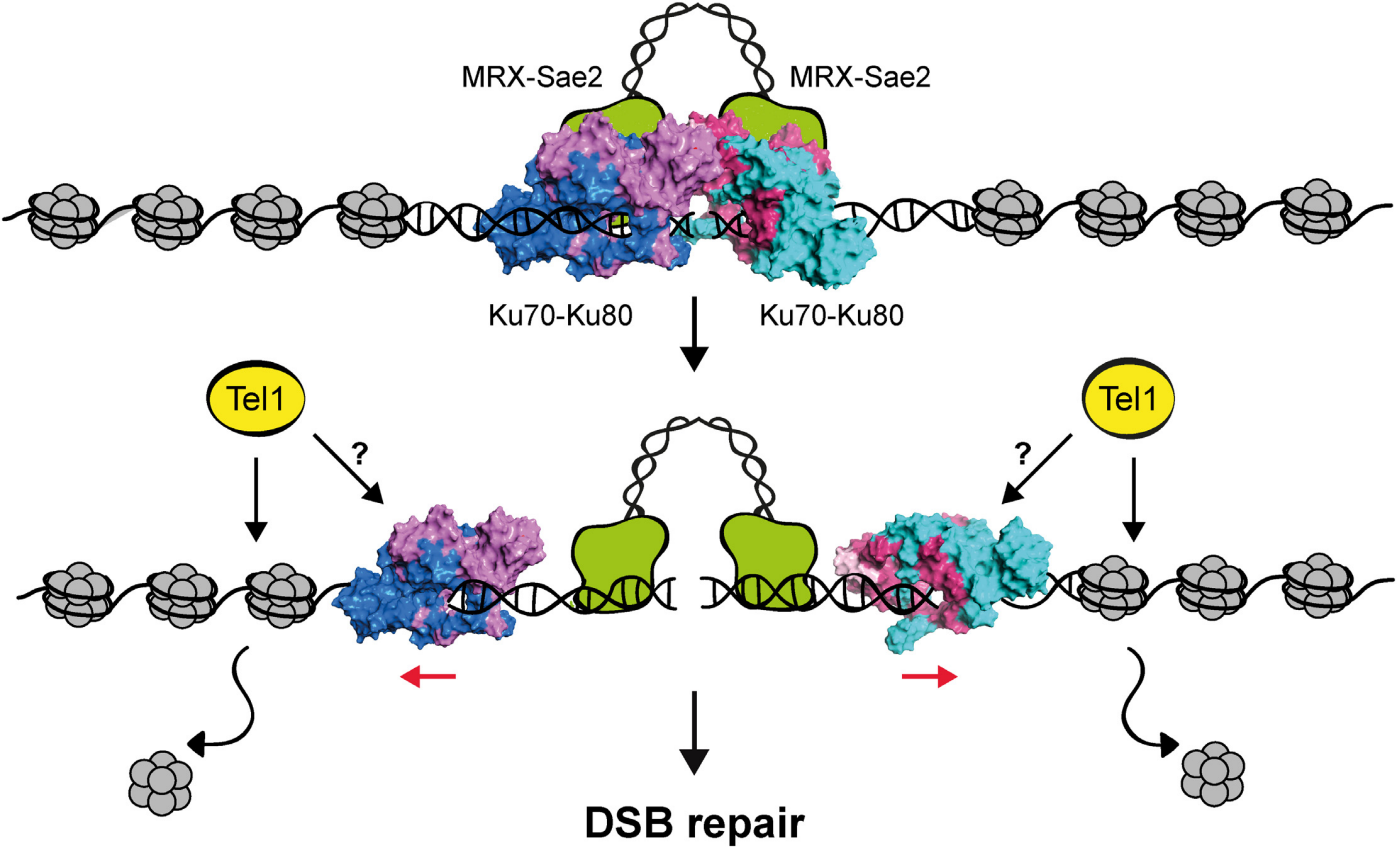
Model for Ku function at DSBs. After DSB formation, two Ku heterodimers are loaded onto both sides of the DSB and contribute to maintain them in close proximity by interacting to each other. This function occurs independently of the MRX complex and Sae2, which also contribute to tether the DSB ends. Tel1 counteracts Ku persistence at the DSB ends by promoting histone removal from DSBs and Ku sliding inwards (red arrows). We cannot exclude the possibility that Tel1 can also act directly on Ku to control its association with DSB. Ku sliding can allow the loading of nucleases that initiate DSB resection and channel DSB repair into HR. The structure of the Ku heterotetramer was built by protein-protein docking simulations, where the interface spans the two vWA-like domains of the Ku70 subunits.

By preventing the broken chromatid from physically separating from the rest of the chromosome, the maintenance of the DSB ends tethered to each other facilitates their correct repair by NHEJ and the homology search during HR. Whether the Ku heterodimer has an *in vivo* role in bridging the DSB ends together has remained somewhat obscure. By characterizing a *ku70* mutation that increases DNA damage resistance of *sae2*Δ cells in a Exo1-independent manner, we provide evidence that the Ku complex has a role in maintaining an intrachromosomal association between the ends of a broken chromosome (Figure 8). In fact, the *ku70-C85Y* allele increases DSB end-tethering and suppresses the end-tethering defect of *sae2*Δ cells. This Ku function in supporting end-bridging occurs independently of MRX, as the *ku70-C85Y* allele also partially suppresses the bridging defects of *mre11*Δ and *rad50-VM* cells. Consistent with a role of Ku in end-tethering, we found that Ku70 can self-associate and the lack of Ku70 exacerbates the end-tethering defect of *sae2*Δ cells.

The maintenance of the DSB ends tethered to each other can be important to repair a DSB by both NHEJ and HR. As *sae2*Δ cells are not defective in DSB repair by NHEJ and *ku70-C85Y* cells show a slight NHEJ defect, it is unlikely that the enhanced end-tethering activity conferred by the *ku70-C85Y* mutation might restore *sae2*Δ DNA damage resistance by increasing the NHEJ efficiency. We have previously shown that the lack of Sae2 impairs DSB repair by both SDSA and SSA (48,72). The *ku70-C85Y* mutation partially suppresses both the SDSA and SSA defects of *sae2*Δ cells, suggesting that a defective end-tethering contributes to the DNA damage sensitivity of *sae2*Δ cells by impairing DSB repair by HR. An essential step in DSB repair by SDSA is that both ends of a DSB are engaged in HR at the same time, thus eliminating the possibility that a single DNA strand invades the template and primes DNA synthesis up to the end of the chromosome through the BIR mechanism. Both Sae2 and MRX have been found to suppress BIR events (104), raising the possibility that these proteins can promote SDSA by coordinating the usage of the two ends of a DSB. It was previously shown that MRX and Sae2 coordinate resection at the two DSB ends (105) and that restoration of synchronous resection in *mre11* mutants decreases DSB repair by BIR (104). Based on the finding that the *ku70-C85Y* mutation restores end-tethering and suppresses the SDSA defect of *sae2*Δ cells, we propose that the maintainance of the DSB ends in close proximity can promote DSB repair by SDSA by contributing to execute synchronous resection of the two DSB ends, thus facilitating the annealing of the displaced strand to the other DSB end. The same mechanism can explain also the suppression of the *sae2*Δ SSA defect.

Interestingly, whereas suppression of *sae2*Δ resection defect by *KU70* deletion results in an increased resistance to CPT and MMS (30–32), suppression of *sae2*Δ end-bridging defect by the *ku70-C85Y* allele also restores resistance to phleomycin. This finding suggests that end-tethering is more important than end-resection to repair phleomycin-induced DNA lesions. Consistent with this hypothesis, we have previously identified *mre11* mutations that increase resistance of *sae2*Δ cells to phleomycin by suppressing their end-tethering but not their resection defect (72). Although phleomycin causes DNA cleavage events as ionizing radiation (IR), the finding that *KU70* deletion suppresses the sensitivity of *sae2*Δ cells to IR but not to phleomycin suggests that the DNA ends generated by these two DNA damaging agents could differ in their nature and/or could be processed differently by the cells. In any case, both end-resection and end-tethering can contribute to increase resistance to CPT that, due to the collision between the replication fork and Topoisomerase I trapped on DNA, can cause single-ended DSBs that can be repaired by sister chromatid recombination (106).

How does Ku70^C85Y^ enhance end-tethering? The C85Y mutation increases the amount of Ku bound at the end of a DSB by enhancing its affinity for DNA, arguing that the increased Ku persistence at DNA ends can account for its better ability to support end-tethering and to inhibit Exo1 association with DSBs. The Ku heterodimer, once bound to a DSB, has been proposed to slide along DNA with an energy-free mechanism (92). We found that the amount of DSB-bound Ku70^C85Y^ is higher than wild-type Ku70 very closely to the DSB end, whereas it decreases with increasing distance from the DSB end. This different Ku enrichment depending on the distance from the DSB ends is consistent with a sliding defect that retains Ku70^C85Y^–Ku80 at the DNA end. The limited Ku70^C85Y^–Ku80 diffusion can be due to the higher affinity of the mutant complex for DNA that imposes a higher energetic barrier to inward movement. In fact, in order to allow energy-free sliding of the Ku dimer along the DNA molecule, the interactions between the protein surface and the DNA have to be easily breakable by mere molecules vibration energy. The more stable interaction established by the Ku70^C85Y^–Ku80 heterodimer with DNA would impose a stronger energetic barrier, making its overrun a rarer event. An increased retention of the Ku70^C85Y^–Ku80 heterodimer on DNA can also limit the accessibility of Exo1 and of other NHEJ proteins to the DSB ends, thus explaining the resection and the NHEJ defects of *ku70-C85Y* cells.

High-resolution microscopy experiments have shown that Ku translocation on DNA appears to be more limited in a cellular context (95), suggesting the existence of mechanisms that suppress Ku diffusion. We found that the lack of Tel1 kinase activity suppresses the end-tethering defect of *sae2*Δ cells. Furthermore, it increases the amount of Ku70 bound very closely to the DSB end, suggesting that Tel1 kinase antagonizes the ability of Ku to support DSB end-tethering by counteracting its persistence at the DSB end. Tel1 kinase might exert this function by regulating Ku conformational changes and/or the activity of proteins that promote or inhibit Ku translocation. Several studies conducted in mammals and yeast support nucleosome eviction in the immediate vicinity of DSB sites with phosphorylated H2AX (γH2AX) being generated in the adjacent chromatin (100,101,107,108). Interestingly, Ku was shown to be less able to load and translocate internally on nucleosome-associated DNA ends (40,98). Furthermore, in mammals Ku and γH2AX foci are mutually exclusive, with Ku foci being flanked by γH2AX (95), suggesting that Ku localizes to DNA ends that are locally depleted of nucleosomes. We found that Tel1 promotes histone disassembly from DSBs. Furthermore, the lack of Isw2 chromatin remodeler, which increases histone persistence at the DSB end, mimics the effect caused by the lack of Tel1 kinase on Ku-mediated DSB tethering and Ku association with DSBs, suggesting that the presence of nucleosomes helps to retain Ku at the DNA end and therefore to promote its function in end-bridging. In any case, although *ISW2* deletion impairs nucleosome removal from DSBs more severely than the lack of Tel1 kinase activity, the *tel1-kd* allele suppresses the end-tethering defect of *sae2*Δ cells more efficiently than *isw2*Δ, suggesting that Tel1, besides removing histones, could act directly on Ku to regulate its DSB association.

In summary, we propose that the Ku heterodimer is loaded on each side of a DSB and contributes to hold the DNA ends together (Figure 8). This function occurs independently of the MRX complex and Sae2, which also support DSB end-tethering. Tel1 kinase counteracts this Ku function by promoting nucleosome removal from DSBs and Ku sliding inwards. As the presence of Ku at the DSB ends prevents the access of resection nucleases, this Tel1-mediated regulation of Ku association with the DSB ends provides an important layer of control in the choice between NHEJ and HR.

## DATA AVAILABILITY

All relevant data are included in the manuscript and the Supplementary Data file. Any other data are available from the authors on request.

## Supplementary Material

gkad062_Supplemental_File
