# Supplementary material for: The Ku complex promotes DNA end-bridging and this function is antagonized by Tel1/ATM kinase
Source: Nucleic Acids Res. 2023 Feb 10;51(4):1783–802. doi: 10.1093/nar/gkad062 (PMC9976877; doi:10.1093/nar/gkad062)
Supplement: gkad062_Supplemental_File [file gkad062_supplemental_file.pdf]

**Table S1.** ***Saccharomyces cerevisiae*** **strains used in this study.**

| Strain | Relevant genotype | Source |
|---|---|---|
| W303 | *MATa/α ade2-1 can1-100 his3-11,15 leu2-3,112 trp1-1 ura3-1 rad5-535* | |
| YLL 1069.3 | W303 *MATa sae2Δ::KANMX* | 1 |
| YLL 4537.3 | W303 *MATa ku70-C85Y::URA3 sae2Δ::KANMX* | This study |
| YLL 4610.8 | W303 *MATa ku70-A90T::URA3 sae2Δ::KANMX* | This study |
| YLL 4611.7 | W303 *MATa ku70-N104Y::URA3 sae2Δ::KANMX* | This study |
| YLL 4612.20 | W303 *MATa ku70-G79S::URA3 sae2Δ::KANMX* | This study |
| YLL 4551.2 | W303 *MATa ku70-D173G::URA3 sae2Δ::KANMX* | This study |
| DMP 7521/1A | W303 *MATα ku70-C85Y::URA3 sae2Δ::KANMX* | This study |
| DMP 7523/2A | W303 *MATα ku70-C85Y::URA3* | This study |
| YLL 4609.4 | W303 *MATa ku70-G79S::URA3* | This study |
| YLL 4535.2 | W303 *MATa ku70-A90T::URA3* | This study |
| YLL 4608.2 | W303 *MATa ku70-N104Y::URA3* | This study |
| DMP 7586/10B | W303 *MATa ku70-D173G::URA3* | This study |
| YLL 941.1 | W303 *MATa ku70Δ::HIS3* | 2 |
| DMP 7541/7B | W303 *MATα ku70Δ::HIS3 sae2Δ::KANMX* | This study |
| DMP 7560/4C | W303 *MATα ku70Δ::HIS3 sae2Δ::KANMX* | This study |
| DMP 7609.1 | W303 *MATa/α KU70-3HA::URA3/KU70-FLAG::KANMX* | This study |
| DMP 7739.1 | W303 *MATa/α ku70-C85Y-3HA::URA3/ku70-C85Y-FLAG::KANMX* | This study |
| DMP 5781/1B | W303 *MATa rad50-V1269M::KANMX* | 3 |
| DMP 7615/1B | W303 *MATa ku70-C85Y::URA3 rad50-V1269M::KANMX* | This study |
| DMP 7648/6D | W303 *MATa exo1Δ::HIS3* | This study |
| DMP 7648/3C | W303 *MATa ku70-C85Y::URA3 exo1Δ::HIS3* | This study |
| DMP 7648/4D | W303 *MATa ku70-C85Y::URA3 sae2Δ::KANMX exo1Δ::HIS3* | This study |
| DMP 7647/6D | W303 *MATa ku70-A90T::URA3 sae2Δ::KANMX exo1Δ::HIS3* | This study |
| DMP 7649/2A | W303 *MATa ku70-N104Y::URA3 sae2Δ::KANMX exo1Δ::HIS3* | This study |
| DMP 7650/1A | W303 *MATa ku70-D173G::URA3 sae2Δ::KANMX exo1Δ::HIS3* | This study |
| DMP 7651/4C | W303 *MATa ku70-G79S::URA3 sae2Δ::KANMX exo1Δ::HIS3* | This study |
| DMP 7652/22B | W303 *MATa sae2Δ::KANMX exo1Δ::HIS3* | This study |
| DMP 7657/4A | W303 *MATa ku70Δ::HIS3 sae2Δ::KANMX exo1Δ::HIS3* | This study |
| DMP 7653/3B | W303 *MATa mre11-H125N* | This study |
| DMP 7641/7C | W303 *MATa ku70Δ::HIS3* | This study |
| DMP 7653/9A | W303 *MATa ku70-C85Y::URA3 mre11-H125N* | This study |

| | | |
|---|---|---|
| DMP 7654/7B | W303 *MATα ku70Δ::HIS3 mre11-H125N* | This study |
| DMP 7660/11A | W303 *MATa ku70-C85Y::URA3 mre11Δ::HIS3* | This study |
| DMP 7660/9B | W303 *MATa mre11Δ::HIS3* | This study |
| DMP 7613/10C | W303 *MATa ku70-C85Y::URA3 sae2Δ::KANMX nej1Δ::HIS3* | This study |
| DMP 7614/10D | W303 *MATa sae2Δ::KANMX nej1Δ::HIS3* | This study |
| DMP 7612/5A | W303 *MATa ku70-C85Y::URA3 nej1Δ::HIS3* | This study |
| DMP 7612/1A | W303 *MATa nej1Δ::HIS3* | This study |
| YLL 4189.3 | W303 *MATa isw2Δ::HIS3* | 4 |
| DMP 7636/5D | W303 *MATa isw2Δ::HIS3 ku70Δ::HIS3 sae2Δ::KANMX* | This study |
| DMP 7642/8C | W303 *MATa isw2Δ::HIS3 sae2Δ::KANMX* | This study |
| DMP 7642/8D | W303 *MATα sae2Δ::KANMX* | This study |
| DMP 7643/8B | W303 *MATa isw2Δ::HIS3 ku70Δ::HIS3* | This study |
| DMP 7731.1 | W303 *MATa/α KU70/KU70 SAE2/SAE2* | This study |
| DMP 7732.1 | W303 *MATa/α KU70/ku70-C85Y::URA3 sae2Δ::KANMX/ sae2Δ::KANMX* | This study |
| DMP 7733.1 | W303 *MATa/α ku70-C85Y::URA3/ku70-C85Y::URA3 sae2Δ::KANMX/ sae2Δ::KANMX* | This study |
| DMP 7734.1 | W303 *MATa/α KU70/KU70 sae2Δ::KANMX/ sae2Δ::KANMX* | This study |
| YLL 4634.3 | W303 *MATa ku70-Y494N::URA3 sae2Δ::KANMX* | This study |
| DMP 7735/9A | W303 *MATa ku70-Y494N::URA3* | This study |
| DMP 7736/14A | W303 *MATa ku70-Y494N::URA3 sae2Δ::KANMX exo1Δ::HIS3* | This study |
| DMP 7737/6B | W303 *MATa ku70-Y494N::URA3 exo1Δ::HIS3* | This study |
| DMP 7738/16D | W303 *MATa ku70-Y494N::URA3 rad50-V1269M::KANMX* | This study |
| JKM 139 | *MATa hmlΔ::ADE1 hmrΔ::ADE1 ade1-100 lys5 leu2-3, 112 trp::hisG ura3-52 ho ade3::GAL-HO site* | 5 |
| YLL 1523.3 | JKM139 *MATa sae2Δ::KANMX* | 1 |
| DMP 7529/2D | JKM139 *MATa ku70-C85Y::URA3* | This study |
| YLL 4548.6 | JKM139 *MATa ku70-C85Y::URA3 sae2Δ::KANMX* | This study |
| DMP 6433/6C | JKM139 *MATa KU70-3HA::URA3 bar1Δ::TRP1* | 6 |
| DMP 7562/2B | JKM139 *MATa ku70-C85Y-3HA::TRP1 bar1Δ::TRP1* | This study |
| DMP 7621/12A | JKM139 *MATa KU70-3HA::URA3 tel1-kd::LEU2 bar1Δ::TRP1* | This study |
| DMP 7622/24A | JKM139 *MATa ku70-C85Y-3HA::URA3 tel1-kd::LEU2 bar1Δ::TRP1* | This study |
| DMP 7638/19D | JKM139 *MATa KU70-3HA::URA3 isw2Δ::HPHMX bar1Δ::TRP1* | This study |
| DMP 6758/8B | JKM139 *MATa EXO1-MYC bar1Δ::HPHMX* | 6 |
| DMP 7655/1A | JKM139 *MATa EXO1-18MYC::TRP1 ku70Δ::URA3 bar1Δ::HPHMX* | This study |
| DMP 7625/4C | JKM139 *MATa EXO1-MYC ku70-C85Y::URA3 bar1Δ::HPHMX* | This study |

| | | |
|---|---|---|
| DMP 6187/3B | JKM139 *MATa tel1-kd::LEU2* | 1 |
| YLL 4264.1 | JKM139 *MATa isw2Δ::HPHMX* | 4 |
| YJK 40.6 | *MATΔ hmlΔ hmrΔ can1 lys5 ade2 leu2 trp1 ura3 his3 ade3:: GAL-HO VII::TRP1-HO LacI-GFP::URA3 LacO::LYS5 LacO::KanR* | 7 |
| YLL 4538.11 | YJK40.6 *ku70-C85Y::LEU2* | This study |
| YLL 1709.11 | YJK40.6 *sae2Δ::NATMX* | 8 |
| YLL 4540.2 | YJK40.6 *ku70-C85Y::LEU2 sae2Δ::NATMX* | This study |
| YLL 4555.4 | YJK40.6 *ku70Δ::LEU2* | This study |
| YLL 4558.1 | YJK40.6 *ku70Δ::LEU2 sae2Δ::NATMX* | This study |
| YLL 1731.29 | YJK40.6 *mre11Δ::NATMX* | 8 |
| YLL 4574.1 | YJK40.6 *ku70-C85Y::LEU2 mre11Δ::NATMX* | This study |
| YLL 3641.6 | YJK40.6 *rad50-V1269M::HPHMX* | 3 |
| YLL 4570.1 | YJK40.6 *ku70-C85Y::LEU2 rad50-V1269M::HPHMX* | This study |
| YLL 4589.4 | YJK40.6 *tel1-kd::LEU2* | This study |
| YLL 4587.2 | YJK40.6 *tel1-kd::LEU2 sae2Δ::NATMX* | This study |
| YLL 4591.2 | YJK40.6 *isw2Δ::LEU2* | This study |
| YLL 4592.11 | YJK40.6 *isw2Δ::LEU2 sae2Δ::NATMX* | This study |
| YLL 4608.3 | YJK40.6 *isw2Δ::HPHMX ku70Δ::LEU2 sae2Δ::NATMX* | This study |
| YLL 4635.4 | YJK40.6 *ku70-Y494N::LEU2* | This study |
| YLL 4636.1 | YJK40.6 *ku70-Y494N::LEU2 rad50-V1269M::HPHMX* | This study |
| tGI354 | *ho hmlΔ::ADE1 MATa-inc hmrΔ::ADE1 ade1 leu2-3;112 lys5 trp1::hisG ura3-52 ade3::GAL::HO arg5,6::MATa::HPHMX* | 9 |
| YLL 3914.2 | tGI354 *sae2Δ::NATMX* | 10 |
| YLL 4637.3 | tGI354 *ku70-C85Y::LEU2* | This study |
| YLL 4638.12 | tGI354 *ku70-C85Y::LEU2 sae2Δ::NATMX* | This study |
| YMV45 | *ho hml::ADE1 mata::hisG hmr::ADE1 leu2::leu2(Asp718-SalI)-URA3*-pBR332-*MATa ade3::GAL::HO ade1 lys5 ura3-52 trp1::hisG* | 11 |
| YLL 1621.9 | YMV45 *sae2Δ::KANMX* | 1 |
| YLL 4639.10 | YMV45 *ku70-C85Y::LEU2* | This study |
| YLL 4640.4 | YMV45 *ku70-C85Y::LEU2 sae2Δ::KANMX* | This study |

## REFERENCES


1 Gobbini,E., Villa,M., Gnugnoli,M., Menin,L., Clerici,M. and Longhese,M.P. (2015) Sae2 function at DNA double-strand breaks is bypassed by dampening Tel1 or Rad53 activity. *PLoS Genet.*, **11**, e1005685.

2 Marsella,A., Gobbini,E., Cassani,C., Tisi,R., Cannavo,E., Reginato,G., Cejka,P. and Longhese,M.P. (2021) Sae2 and Rif2 regulate MRX endonuclease activity at DNA double-strand breaks in opposite manners. *Cell Rep.*, **34**, 108906.

3 Cassani,C., Gobbini,E., Wang,W., Niu,H., Clerici,M., Sung,P., and Longhese,M.P. (2016) Tel1 and Rif2 regulate MRX functions in end-tethering and repair of DNA double-strand breaks. *PLoS Biol.*, **14**, e1002387.

4 Casari,E., Gobbini,E., Gnugnoli,M., Mangiagalli,M., Clerici,M. and Longhese,M.P. (2021) Dpb4 promotes resection of DNA double-strand breaks and checkpoint activation by acting in two different protein complexes. *Nat. Commun.*, **12**, 4750.

5 Lee,S.E., Moore,J.K., Holmes,A., Umezu,K., Kolodner,R.D. and Haber,J.E. (1998) *Saccharomyces* Ku70, Mre11/Rad50 and RPA proteins regulate adaptation to G2/M arrest after DNA damage. *Cell*, **94**, 399-409.

6 Gobbini,E., Cassani,C., Vertemara,J., Wang,W., Mambretti,F., Casari,E., Sung,P., Tisi,R., Zampella,G. and Longhese,M.P. (2018) The MRX complex regulates Exo1 resection activity by altering DNA end structure. *EMBO J.*, **37**, e98588.

7 Kaye,J.A., Melo,J.A., Cheung,S.K., Vaze,M.B., Haber,J.E. and Toczyski,D.P. (2004) DNA breaks promote genomic instability by impeding proper chromosome segregation. *Curr. Biol.*, **14**, 2096-2106.

8 Clerici,M., Mantiero,D., Lucchini,G. and Longhese,M.P. (2005) The *Saccharomyces cerevisiae* Sae2 protein promotes resection and bridging of double strand break ends. *J. Biol. Chem.*, **280**, 38631-38638.

9 Saponaro,M., Callahan,D., Zheng,X., Krejci,L., Haber,J.E., Klein,H.L. and Liberi,G. (2010) Cdk1 targets Srs2 to complete synthesis-dependent strand annealing and to promote recombinational repair. *PLoS Genet.*, **6**, e1000858.

10 Cassani,C., Gobbini,E., Vertemara,J., Wang,W., Marsella,A., Sung,P., Tisi,R., Zampella,G. and Longhese,M.P. (2018) Structurally distinct Mre11 domains mediate MRX functions in resection, end-tethering and DNA damage resistance. *Nucleic Acids Res.*, **46**, 2990-3008.

11 Vaze,M.B., Pellicioli,A., Lee,S.E., Ira,G., Liberi,G., Arbel-Eden,A., Foiani,M. and Haber,J.E. (2002) Recovery from checkpoint-mediated arrest after repair of a double-strand break requires Srs2 helicase. *Mol. Cell*, **10**, 373-385.

**Table S2. Oligonucleotides used for gene tagging and disruptions.**

| Name | Sequence (5'-3') |
|---|---|
| PRP171 | CAATAGTGGAGAACTTAACGATCAAGTGGATGAAACAGGTTATACGT ACGCTGCAGGTCGAC |
| PRP172 | ATTTAGCCTTTGGATGATTGGATCTTCTGACTTCTCAGATTCTTAATCG ATGAATTCGAGCTCG |
| PRP330 | TGAATAAAGAATGATGATCGCTGGCGTTTAACATCTAGCATATATCTG CAATAATTTATCACTCATCGATGAATTCGAGCTCG |
| PRP331 | TGTAAGCCATTAGGTGTTTGTATGTGAGATGGTGACTGGTGAAGAAAA TGTGTATCTAAAGCGTACGCTGCAGGTCGAC |
| PRP414 | AGATAGGAGGTTAATCTTACACATGTGTGATGGATTCTGAGTTGACGT ACGCTGCAGGTCGAC |
| PRP415 | AATTTTTGACGTTTAATTGACTTGCCTTAGTTTTTATTCTCACCATCGA TGAATTCGAGCTCG |
| PRP763 | TTTAACGATGATAACATTTCGATAAAAGAAGAAAAGAAGCCCTTTGAT AAAAAGCCGAAATTCAATATATCCGGTTCTGCTGCTAG |
| PRP764 | TACATCAAATACCCTACCCTACCAAATATTGTATGTAACGTTATAGATA TGAAGGATTTCAATCGTCTCCTCGAGGCCAGAAGAC |
| PRP783 | GTCAAGCCAAGCTCGGCCGGCAGTCAGATCTATCTCCTTGCTTTCCCA ATTTGTTTATAAAGGTAAATCCGGTTCTGCTGCTAG |
| PRP784 | GAGGCATTTCGACGAGATTTTCATTTGAAAAATATACCTCCGATATGA AACGTGCAGTACTTAACTTCCTCGAGGCCAGAAGAC |
| PRP2284 | AATATAGATTCCATTTTGAGATTTCTATCCTCGAGGAGAACTTCTAATA TTGAAAGAGAGAGAGAGAAGC |
| PRP2285 | GTTGGGATTGCATTGTTGATCAAGGCTATATAATATTATGTACACAGA ATGTCGTTCATGGTGACACTTT |
| PRP2506 | AATATAGATTCCATTTTGAGATTTCTATCCTCGAGGAGAACTTCTAATA TTGCAGGCTAACCGGAACCTG |
| PRP2507 | GTTGGGATTGCATTGTTGATCAAGGCTATATAATATTATGTACACAGA ATCATTGGCCCTACCCACATATG |
| PRP2541 | CAATAGTGGAGAACTTAACGATCAAGTGGATGAAACAGGTTATATGC AGGCTAACCGGAACCTG |
| PRP2542 | ATTTAGCCTTTGGATGATTGGATCTTCTGACTTCTCAGATTCTACATTG GCCCTACCCACATATG |
| PRP2577 | TTTAACGATGATAACATTTCGATAAAAGAAGAAAAGAAGCCCTTTGAT AAAAAGCCGAAATTCAATATATCCCACCACCATCATCATCAC |
| PRP2578 | TACATCAAATACCCTACCCTACCAAATATTGTATGTAACGTTATAGATA TGAAGGATTTCAATCGTCTACTATAGGGAGACCGGCAGATC |
| PRP2712 | AGGAAAACTTACAATCAGATCATGACGACCCAGCAAGAGCATGCAGG CTAACCGGAACCTG |
| PRP2713 | CTCTCACGTCACTTATTTTAATGCACAATACATGATTCATGCCATTGGC CCTACCCACATATG |

**Table S3. Oligonucleotides used for qPCR.**

| **Name** | **Sequence (5'-3')** |
|---|---|
| ARO+ | TGAGTCGTTACAAGGTGATGCC |
| ARO- | ACCTACAGGAGGACCCGAAA |
| DSB 0.2+ | TCAGACTCAAGCAAACAATCAA |
| DSB 0.2- | CCCGTATAGCCAATTCGTTC |
| DSB 0.6+ | CACCCAAGAAGGCGAATAAG |
| DSB 0.6- | CATGCGGTTCACATGACTTT |
| DSB 1.8+ | ACGTCGTTGTTAATGGTGGTG |
| DSB 1.8- | CGCGAGTCTTATGCCAAAAA |

**Figure S1**

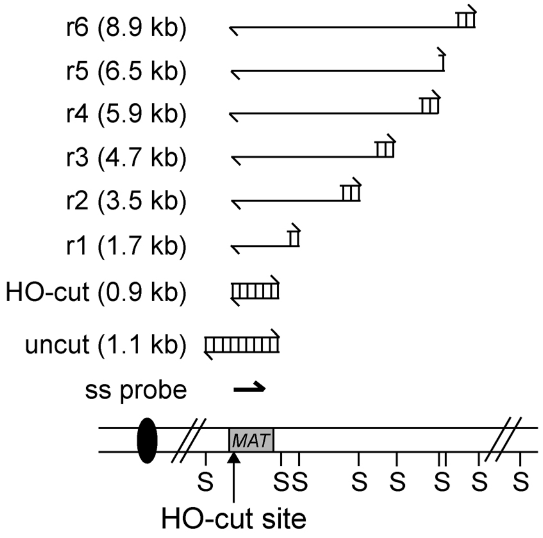

r6 (8.9 kb)
r5 (6.5 kb)
r4 (5.9 kb)
r3 (4.7 kb)
r2 (3.5 kb)
r1 (1.7 kb)
HO-cut (0.9 kb)
uncut (1.1 kb)
ss probe
MAT
S
SS
S
S
S
S
S
HO-cut site

**Figure S2**

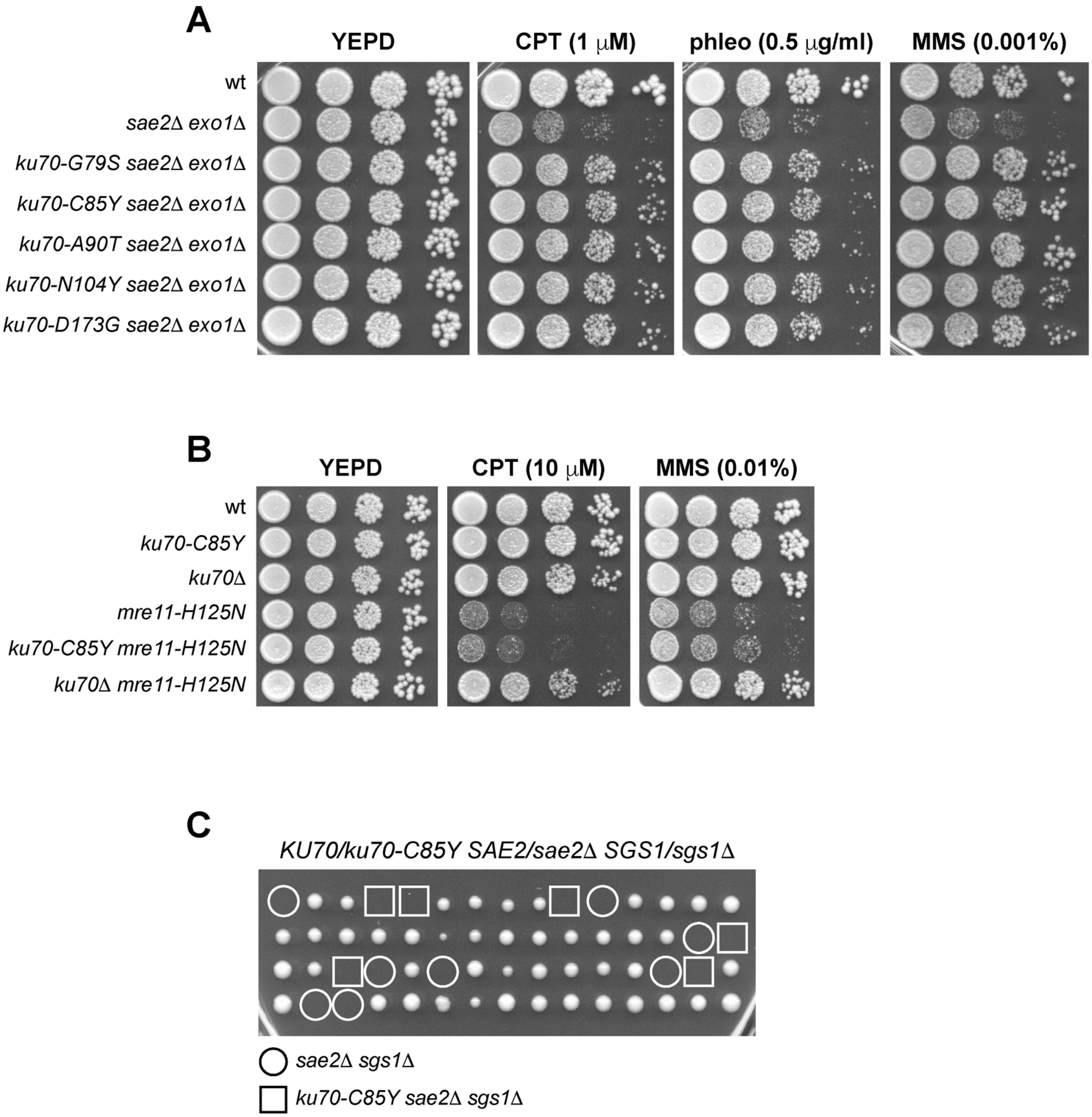
A
YEPD
CPT (1 μM)
phleo (0.5 μg/ml)
MMS (0.001%)
wt
sae2Δ exo1Δ
ku70-G79S sae2Δ exo1Δ
ku70-C85Y sae2Δ exo1Δ
ku70-A90T sae2Δ exo1Δ
ku70-N104Y sae2Δ exo1Δ
ku70-D173G sae2Δ exo1Δ
B
YEPD
CPT (10 μM)
MMS (0.01%)
wt
ku70-C85Y
ku70Δ
mre11-H125N
ku70-C85Y mre11-H125N
ku70Δ mre11-H125N
C
KU70/ku70-C85Y SAE2/sae2Δ SGS1/sgs1Δ
sae2Δ sgs1Δ
ku70-C85Y sae2Δ sgs1Δ

# Figure S3

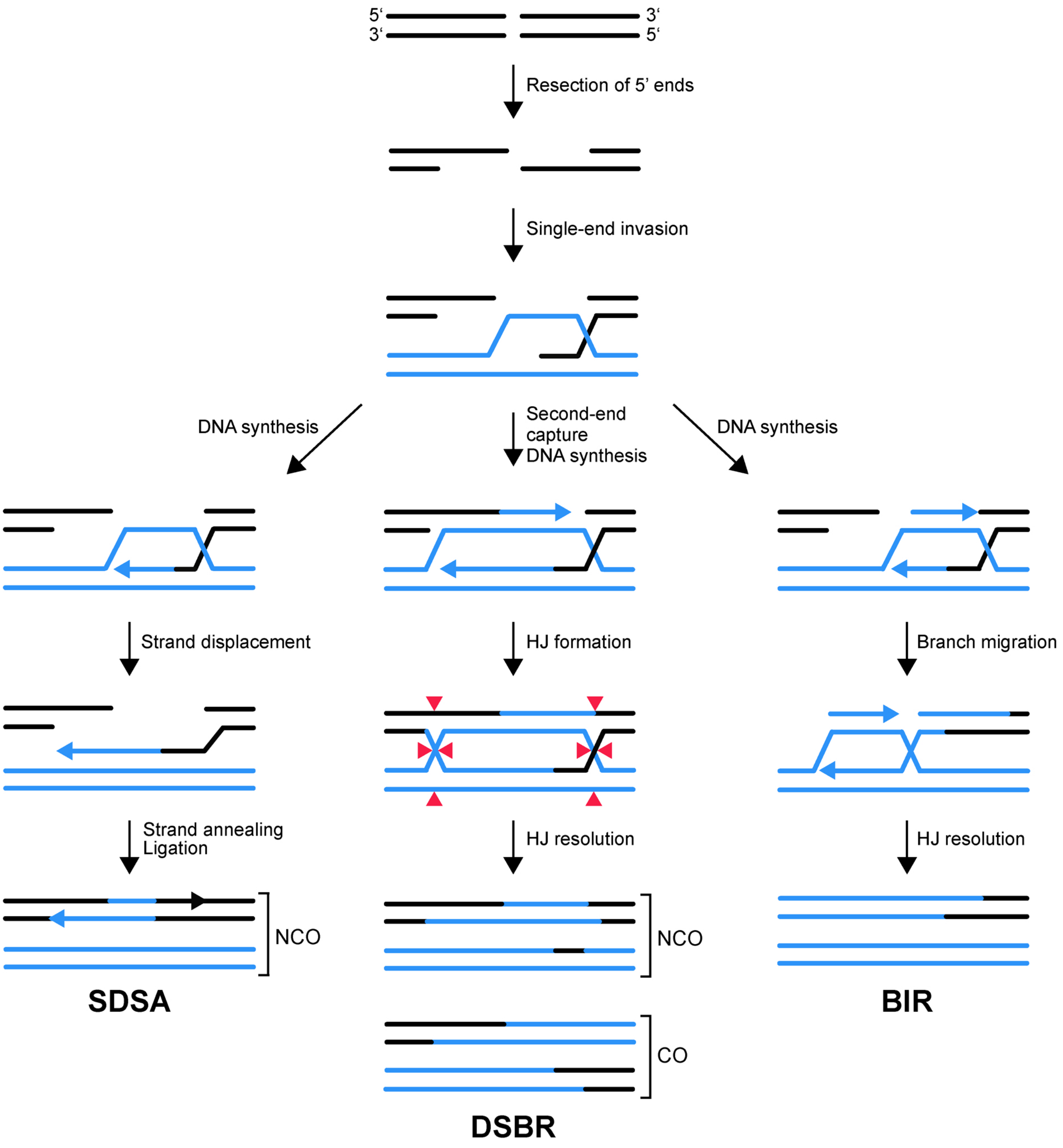
5'
3'
3'
5'
Resection of 5' ends
Single-end invasion
DNA synthesis
Second-end capture
DNA synthesis
DNA synthesis
Strand displacement
HJ formation
Branch migration
Strand annealing
Ligation
HJ resolution
HJ resolution
NCO
SDSA
NCO
CO
DSBR
BIR

**Figure S4**

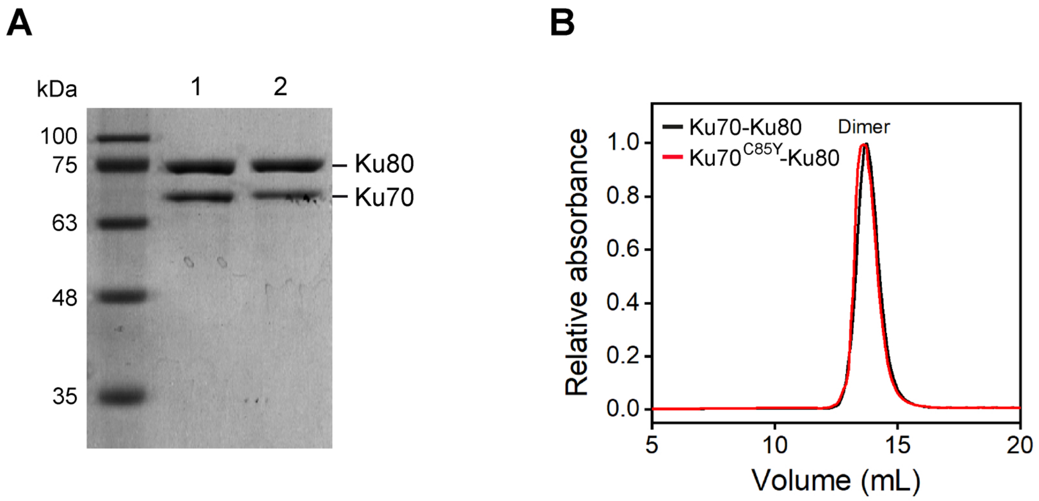
A
kDa
1
2
100
75
63
48
35
Ku80
Ku70
B
Ku70-Ku80
Ku70^C85Y-Ku80
Dimer
Relative absorbance
1.0
0.8
0.6
0.4
0.2
0.0
5
10
15
20
Volume (mL)

# Figure S5

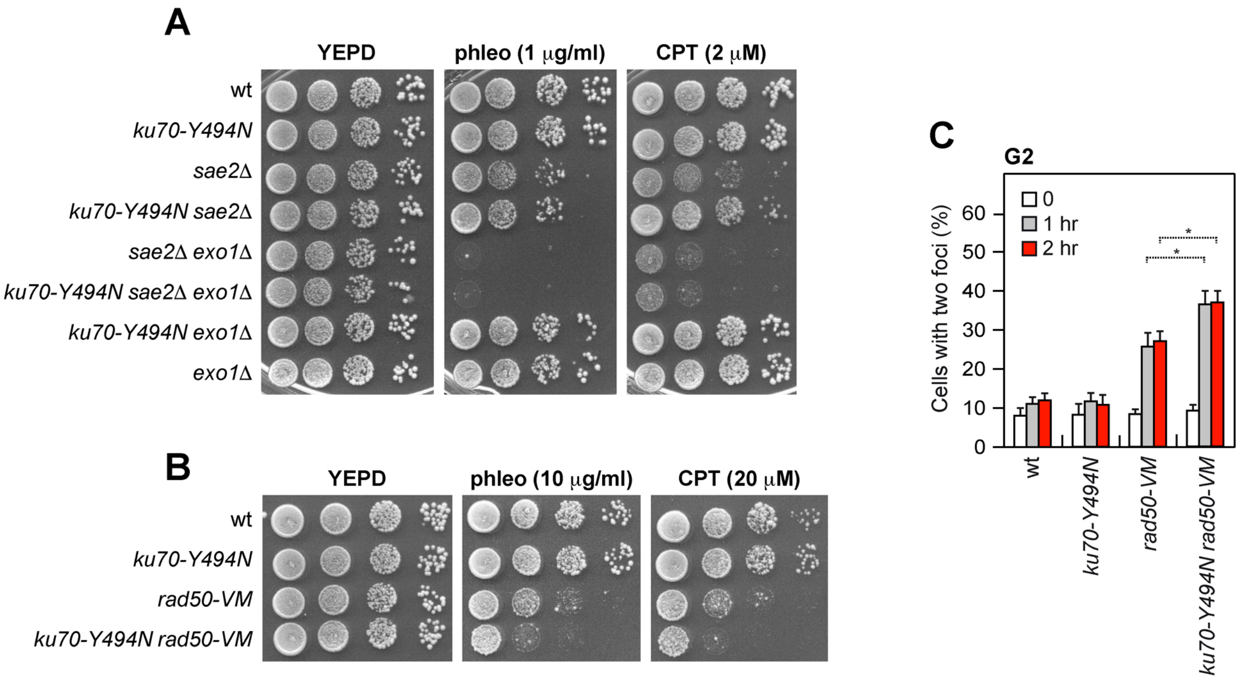
A
YEPD
phleo (1 μg/ml)
CPT (2 μM)
wt
ku70-Y494N
sae2Δ
ku70-Y494N sae2Δ
sae2Δ exo1Δ
ku70-Y494N sae2Δ exo1Δ
ku70-Y494N exo1Δ
exo1Δ
B
YEPD
phleo (10 μg/ml)
CPT (20 μM)
wt
ku70-Y494N
rad50-VM
ku70-Y494N rad50-VM
C
G2
Cells with two foci (%)
0
1 hr
2 hr
*
*
60
50
40
30
20
10
0
wt
ku70-Y494N
rad50-VM
ku70-Y494N rad50-VM

## SUPPLEMENTARY FIGURE LEGENDS

**Figure S1.** System used to detect DSB resection. Resection of the DSB end progressively eliminates *Ssp*I sites (S), producing longer *Ssp*I fragments (r1 through r6) that can be separated on an alkaline agarose gel and visualized after hybridization with a single-strand RNA probe that anneals to the unresected strand at one side of the DSB.

**Figure S2.** Effects of the *ku70* alleles on the DNA damage sensitivity of *sae2*Δ *exo1*Δ and *mre11-H125N* cells and on viability of *sae2*Δ *sgs1*Δ cells. (**A,B**) Exponentially growing cultures were serially diluted (1:10) and each dilution was spotted out onto YEPD plates with or without camptothecin (CPT), phleomycin (phleo) or methyl methanesulfonate (MMS). (**C**) Meiotic tetrads were dissected on YEPD plates that were incubated at 25°C, followed by spore genotyping.

**Figure S3.** Models for DSB repair by SDSA, classical DSB repair (DSBR) and BIR. Lines represent single DNA strands and arrowheads indicate 3' ends of the newly synthesized strands. In the canonical DSBR pathway (center), cleavage of the two HJ intermediates in the same direction (both horizontal or both vertical junctions) generates a noncrossover (NCO) product, while cleavage in different directions leads to a crossover (CO) product. During SDSA (left), only a single end interacts with the repair template and only NCOs are generated. During BIR (right), invasion of the repair template is followed by conservative DNA synthesis that restores the end of the broken DNA.

**Figure S4.** Purification of Ku70-Ku80 and Ku70$^{C85Y}$-Ku80 complexes. (**A**) SDS-PAGE analysis of purified Ku70-Ku80 (lane 1) and Ku70$^{C85Y}$-Ku80 (lane 2) complexes. (**B**) Quaternary structure of Ku70-Ku80 and Ku70$^{C85Y}$-Ku80 complexes determined by SEC analysis. One of three independent measurements was shown. The molecular weight estimated by SEC is 164.93 ± 4.56 kDa and 153.20 ± 5.71 kDa for Ku70-Ku80 and Ku70$^{C85Y}$-Ku80 complexes, respectively.

**Figure S5.** The *ku70-Y494N* allele exacerbates the DNA damage sensitivity and the end-tethering defect of *rad50-VM* cells. (**A,B**) Exponentially growing cultures were serially diluted (1:10) and each dilution was spotted out onto YEPD plates with or without phleomycin or CPT. (**C**) DSB end-tethering. Exponentially growing YEPR cell cultures were arrested in G2 with nocodazole at time zero and transferred to YEPRG in the presence of nocodazole. 200 cells for each strain were analyzed to determine the percentage of cells showing two LacI-GFP foci. The mean values of three independent experiments are represented with error bars denoting s.d. *$p < 0.05$ (unpaired two-tailed Student's *t*-test).
